# Preterm birth buccal cell epigenetic biomarkers to facilitate preventative medicine

**DOI:** 10.1038/s41598-022-07262-9

**Published:** 2022-03-01

**Authors:** Paul Winchester, Eric Nilsson, Daniel Beck, Michael K. Skinner

**Affiliations:** 1 Department of Pediatrics, St. Franciscan Hospital, School of Medicine, Indiana University, Indianapolis, IN 46202-5201 USA; 2 Center for Reproductive Biology, School of Biological Sciences, Washington State University, Pullman, WA 99164-4236 USA

**Keywords:** Developmental biology, Biomarkers, Molecular medicine

## Abstract

Preterm birth is the major cause of newborn and infant mortality affecting nearly one in every ten live births. The current study was designed to develop an epigenetic biomarker for susceptibility of preterm birth using buccal cells from the mother, father, and child (triads). An epigenome-wide association study (EWAS) was used to identify differential DNA methylation regions (DMRs) using a comparison of control term birth versus preterm birth triads. Epigenetic DMR associations with preterm birth were identified for both the mother and father that were distinct and suggest potential epigenetic contributions from both parents. The mother (165 DMRs) and female child (136 DMRs) at *p* < 1e−04 had the highest number of DMRs and were highly similar suggesting potential epigenetic inheritance of the epimutations. The male child had negligible DMR associations. The DMR associated genes for each group involve previously identified preterm birth associated genes. Observations identify a potential paternal germline contribution for preterm birth and identify the potential epigenetic inheritance of preterm birth susceptibility for the female child later in life. Although expanded clinical trials and preconception trials are required to optimize the potential epigenetic biomarkers, such epigenetic biomarkers may allow preventative medicine strategies to reduce the incidence of preterm birth.

## Introduction

Preterm birth (PTB) is childbirth that occurs at less than 37 weeks of gestation. Worldwide, preterm birth rates are estimated at 11%, accounting for about 14.8 million of the live births of 2014^1^. Complications of being born preterm were the leading cause of mortality in children less than five years of age in 2015^2^. Children who survive preterm birth are at increased risk of developing future adverse health outcomes, including cognitive disabilities, seizures, visual and hearing impairment, and cardiovascular problems^3–11^. Although there are many risk factors associated with preterm birth including genetic variants, exposure to environmental toxicants, presence of multiple fetuses, preeclampsia and ethnicity, more than half of premature birth cases have an unknown etiology^9,10,12,13^. Reliable biomarkers for preterm birth could greatly help in predicting which pregnancies are at risk and would improve clinical management and health outcomes for the children.

A number of potential biomarkers for preterm birth have been identified. Maternal serum levels of alpha-fetoprotein (ms-AFP) and human chorionic gonadotropin (ms-hCG) have been used clinically^6–8^. Although many associations between mid-trimester ms-hCG and/or ms-AFP levels and adverse pregnancy outcomes are statistically significant, the sensitivity and positive predictive value are too low for them to be clinically useful as screening tests for preterm birth^3,14^. Other proposed biomarkers of PTB risk include selected inflammatory cytokines^15–18^, metabolic lipid products^17,19^, specific gene mRNA transcripts^20,21^, cervicovaginal proteome^22^, and micro-RNA transcripts^20,23,24^. Urinary oxidative stress metabolites have also been proposed as biomarkers of preterm birth^25,26^. These biomarkers are not extensively used and are not considered efficient or ideal^27^. Either the assays for proteins and metabolites are technically challenging and expensive, or the specificity and sensitivity of the assays in predicting preterm birth need to be improved^27^.

Previous studies have proposed that epigenetic alterations should be considered for use as biomarkers to predict preterm birth^28–31^. Epigenetics is defined as “molecular factors and processes around DNA that regulate genome activity, independent of DNA sequence, and that are mitotically stable”^32^. Epigenetic factors and processes include DNA methylation, histone modifications, non-coding RNA, and chromatin structure changes^33^. Assays for DNA methylation have the advantage of using smaller sample size due to high sensitivity of the assays, as well as being less expensive and technically demanding than assays for proteins and metabolic products. DNA methylation changes can also be detected in easily obtained surrogate samples (i.e., marker cells not directly associated with the etiology of the pathology), such as cheek buccal epithelial cells^34^. This is due to the fact that epigenetic differences can be heritable, so all somatic cells derived from the embryo of an individual have cell-specific epigenetic changes derived from the germline^33^. Altered DNA methylation sites caused by fetal toxicant exposure, abnormal nutrition, or stress have been found in previous studies to be associated with increased risk of disease in exposed offspring and their descendants (i.e., epigenetic transgenerational inheritance)^35–37^.

There is evidence that epigenetic differences are associated with preterm birth in the placenta^38^ and tissues of children born preterm. Studies that compared DNA methylation in umbilical cord blood between preterm and full-term children found from 31 to 296 differentially methylated sites^38–40^. One study found DNA methylation differences in umbilical cord tissue between preterm and full-term children^39^. These results indicate that DNA methylation changes may occur with preterm birth and suggest that DNA methylation changes are worth investigating as a viable biomarker for predicting preterm birth. Although all cell types have the same DNA sequence present, a limitation of examining DNA methylation changes in a mixed cell population, such as blood with over 20 different cell types, is that each cell type has a unique epigenome and DNA methylation profile driving the cell type specificity^32^. Thus, small changes in the relative numbers of different cell types in a mixed population can suggest an epigenetic difference, but are in fact due to the changes in cell population numbers^32,33^. Therefore, purified individual cell types are preferred to effectively assess epigenetic differences and potential disease biomarkers^41,42^.

Changes in DNA methylation at particular genomic loci have been reported as biomarkers associated with human diseases. Sperm samples from men with idiopathic infertility (i.e*.* infertility from no known cause, and not related to low sperm count or motility) were found to have 217 differential DNA methylation regions (DMRs) at a *p* value of *p* < 1e−05 compared to sperm samples from fertile men^43^. In addition, 56 DMRs were found between initially infertile men who responded to follicle stimulating hormone (FSH) therapy versus those who did not, suggesting that DNA methylation may be used as a biomarker of responsiveness to this therapy^43^. Recently it was reported that a set of 805 DMRs in sperm was potentially associated with men having an increased risk of having a child with autism^44^. Previously, it has been shown that DNA methylation at the SLC9B1 gene in blood samples from pregnant women between 24 and 32 weeks gestation can predict whether the fetus is at risk for fetal intolerance of labor, which can cause fetal hypoxia, and is an indication for performing a Caesarean section^45^. In a recent study, we have used buccal cells as an easily obtained purified cell population to identify epigenetic (i.e., DNA methylation) biomarkers for female rheumatoid arthritis^46^. Although sperm epigenetic biomarkers reflect epigenetic inheritance of disease in offspring and subsequent generations, a surrogate cell such as buccal cells can reflect early embryo impacts on all somatic cells to be used for disease assessment^46,47^. Together, these studies indicate that epigenetic biomarkers of preterm birth susceptibility or pathology potentially exist and are worthy of further development. Identification of maternal biomarkers associated with preterm birth could help in the prediction and clinical management of at-risk pregnancies and allow for better preventative care for preterm birth children. Clinical management protocols that could be used to reduce the incidence of preterm birth and infant morbidity include: enhanced surveillance of at-risk pregnancies, timely use of prenatal steroids and tocolytics, application of protective uterine monitoring, hospitalization and operative delivery. Epigenetics may also point the way to specific gene targets for future pharmaceutical agents where epigenetically identified “at risk” women could be given gene-specific therapeutics.

The current study was designed to develop epigenetic biomarkers for preterm birth that could be used in a clinical setting to predict preterm birth susceptibility. Buccal cells were obtained from the mother, father, and child from control (> 37 week gestation) and premature (< 37 week gestation) populations and compared. The goal was to find in maternal and paternal buccal cells DMRs which could distinguish preterm from term birth. Clearly the infant epigenetic biomarker is not used to predict potential preterm birth, but can potentially be used to assess later life disease susceptibility in the individual. These epigenetic biomarkers identified can now be prospectively tested for their positive and negative predictive power in subsequent investigations. The generational study presented suggests potential epigenetic inheritance aspects for preterm birth.

## Results

The objective of the study was to develop an epigenetic (i.e., DNA methylation) biomarker for preterm birth (PTB). One of the least invasive and easiest purified cell types to collect is a buccal swab from the cheek, which is greater than 90% pure squamous epithelial cells^48^. Any contaminating bacterial molecular data can be removed during the analysis. Buccal cells were obtained from participants with a home collection swab kit and sent directly to the lab for storage and analysis. The participants were recruited prior to collection or analysis from Indiana University (IU) Health Hospitals (Riley Hospital for Children, IUH Methodist, IUH North) and Franciscan Health, Indianapolis, Indiana. Approvals to conduct the study were obtained from Indiana University Institutional Review Board (IRB) #1901985132 and the Franciscan Institutional Review Board (IRB), #1489434-5. Informed consent and HIPAA authorization was obtained from all participants and from a parent and/or guardian for participants that were minors prior to the clinical sample collection. The buccal cells were collected from the mother, father, and newborn child (triads) to assess epigenetic biomarkers in each group separately. The triad samples were collected, approximately nine days following delivery. This period was used to allow the PTB case child to mature and allow an effective buccal cell collection. The full term (FT) birth controls had 21 triad participants and the pre-term birth (PTB) cases had 19 triad participants. Although the majority were of non-Hispanic white Caucasian backgrounds, a number of triads in each population were of African American descent, Supplemental Table S1. The presence of the African American participants did not appear to affect the analysis and similar methylation data was observed in these samples, as assessed with a principal component analysis (PCA), Supplemental Figure S1. The samples were collected in 2019 and early 2020, Supplemental Table S1. The mean maternal age was 28.1 years (controls) and 28.7 years (PTB cases) and mean paternal age 30.8 years (controls) and 30.4 years (PTB cases) with no statistical difference between the control or PTB case groups, with no statistical difference between the groups, Supplemental Table S1. The newborn gestational age at birth, mean ± SD was 38.8 ± 0.94 weeks for the control group and 30.2 ± 3.24 weeks for the PTB case group, with statistical difference (*p* ≤ 0.001), Supplemental Table S1. The Supplemental Table S1B presents the clinical demographics for the populations. The preterm pregnancies were found to be significantly more likely to be multiparous and less likely to be primiparous. Therefore, PTB occurrences were more likely to have had one or more of the following clinical conditions: (1) to have had a previous preterm birth or pregnancy loss; (2) more likely to have preeclampsia; (3) to have a medically indicated delivery; and/or (4) to have a delivery accompanied by fetal distress and lower APGAR scores. Preterm infants naturally would have had lower birth weights, shorter gestation, and longer hospital stay. Other maternal characteristics were not significantly different between groups (i.e., maternal age, paternal age, BMI, insurance source, substance use, diabetes, thyroid placental disorders, cervical disorders, infections, neuropsychiatric disorders), Supplemental Table S1B. Since there were no major outliers in the PCA analysis, the various clinical parameters within the PTB group appear not to be variables for the DMRs, but expanded studies are required to thoroughly assess, Supplemental Figure S1. Buccal cells were collected from each group as outlined in the Methods. All samples were stored at − 80 °C until DNA preparation and analysis.

**Figure 1:**
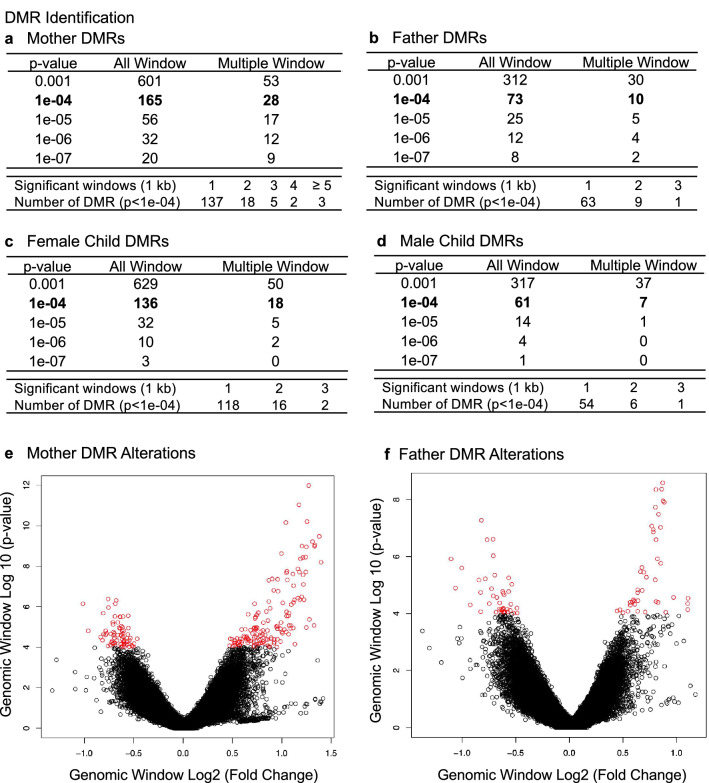
DMR identification and numbers. The number of DMRs found using different p-value cutoff thresholds. The All-Window column shows all DMRs. The Multiple Window column shows the number of DMRs containing at least two nearby significant windows (1 kb each). The number of DMRs with the number of significant windows (1 kb per window) at a *p* value threshold of *p* < 1e−04 for DMR is bolded. (**a**) Mother DMRs; (**b**) Father DMRs; (**c**) Female child DMRs; (**d**) Male child DMRs; (**e**) Mother; and (**f**) Father log-fold-change DMR alterations. The red circles are statistically significant DMRs showing log-fold change distribution (i.e., increase or decrease DNA methylation).

**Figure 2:**
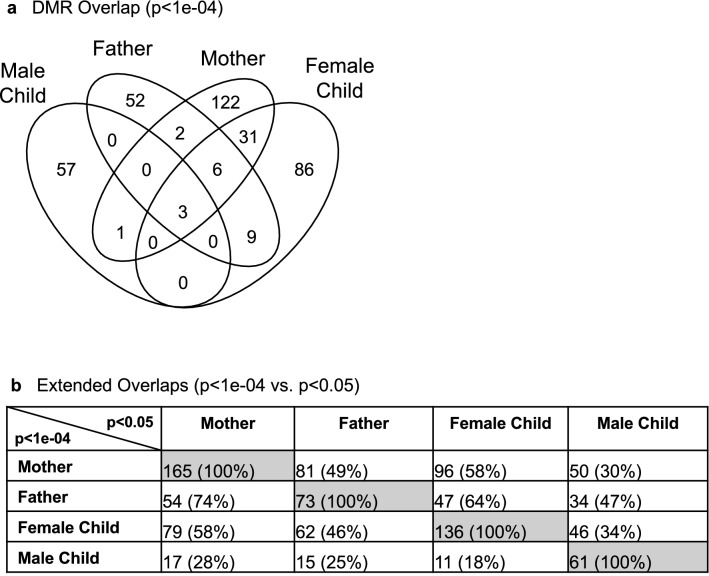
DMR group overlaps. (**a**) DMR *p* < 1e−04 Venn diagram overlap. (**b**) Extended overlaps with *p* < 1e−04 and *p* < 0.05 comparisons. DMR number and percent (%) overlap presented within the rows.

DNA was isolated from the buccal cell collections and analyzed with a methylated DNA immunoprecipitation (MeDIP) procedure to obtain methylated DNA for subsequent sequencing (Seq) for an MeDIP-Seq protocol^49^, as described in the Methods. This procedure can provide a genome-wide assessment of greater than 90% of the genome, compared to approximately 50–70% for bisulfite sequencing or less than 1% for array analysis^50^. Differential DNA methylation regions (DMRs) were identified by comparing the control and PTB case samples for each mother, father, or child triad. DMRs identified were obtained for each group and presented in Fig. 1a for the mother, Fig. 1b for the father, Fig. 1c for the female child, and Fig. 1d for the male child. The DMRs at various edgeR p-value statistical thresholds are presented, and *p* < 1e−04 was used for all subsequent data analysis, which was selected as it also provided a reasonable false discovery rate (FDR). The number of adjacent DMR 1 kb windows are shown at a significance level of *p* < 1e−04 and the majority of DMR for each group had a single 1 kb window with some higher numbers of significant adjacent windows, Fig. 1a–d. Maternal buccal cells had 165 DMRs, paternal 73 DMRs, female child 136 DMRs, and male child 61 DMRs. The FDR p-value was less than 0.1 for 100% of the mother DMRs, 75% for the father DMRs, 50% for the female child, and 25% (i.e., 14 DMRs) for the male child. Therefore, the male child had less significant DMRs, Fig. 1d. Approximately 50% of DMRs showed an increase and 50% a decrease in DNA methylation in each group, Fig. 1e and f and Supplemental Figure S2. An overlap of the DMRs demonstrated each group was primarily distinct at *p* < 1e−04, except for the mother and female child, which shared 31 DMRs in common, Fig. 2a. Further analysis of potential overlaps used an extended overlap analysis with a comparison of the *p* < 1e−04 DMRs with the other groups at a *p* < 0.05 threshold. This extended overlap demonstrated much higher levels of overlaps with maternal DMRs having a 49% overlap with the paternal, 58% with the female child, and 30% with the male child. Paternal DMRs had a 75% overlap with the mother, 64% with the female child, and 47% with the male child. The female child overlaps were higher and ranged from 34 to 58%, while the male child overlap ranged from 18 to 28%, Fig. 2b. Therefore, preterm birth DMR were identified in the buccal cells of the mother and father, as well as in the female children following a preterm birth.

**Figure 3:**
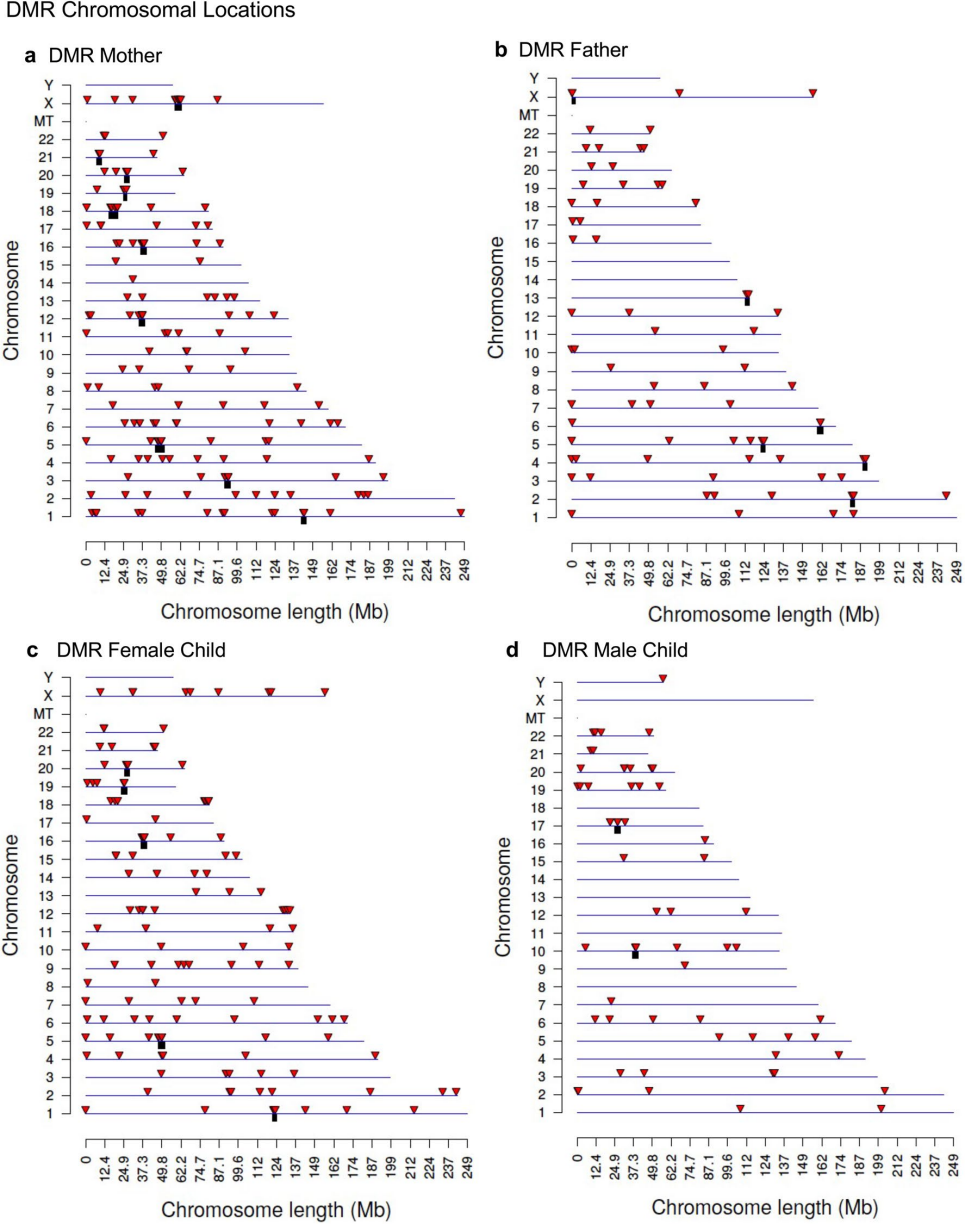
DMR chromosomal locations. The DMR locations on the individual chromosomes is represented with an arrowhead and a cluster of DMRs with a black box. All DMRs containing at least one significant window at a *p* value threshold of *p* < 1e−04 for DMR are shown. (**a**) Mother DMRs; (**b**) Father DMRs; (**c**) Female child DMRs; and (**d**) Male child DMRs. The chromosome number versus size (megabase) is presented.

The lists of DMRs and genomic information are presented in Supplemental Table S2 for the mother, Supplemental Table S3 for the father, Supplemental Table S4 for the female child, and Supplemental Table S5 for the male child. These tables present for each group the DMR name, chromosomal location, start and stop nucleotide number, statistics information (*p* value and FDR), log-fold methylation change (increase positive or decrease negative) for each DMR, gene associations (within 10 kb of gene) and functional categories for the associated genes. The chromosomal locations of the DMRs (red arrowheads) for each group are presented in Fig. 3. The DMRs are present on most chromosomes throughout the genome. The black boxes indicate clusters of DMRs at similar regions. Although some individual DMR overlaps at a 1 kb level are observed, Fig. 2, no obvious gross (Mb size) chromosomal regions or sites are in common between the mother, father or female child genomes, Fig. 3. The size of the DMRs for each group is 1 or 2 kb with a CpG density less than 3 CpG/100 bp, Supplemental Figure S3. These regions with low CpG density are considered CpG desserts^51^, which represents the majority (> 90%) of the genome, but some DMRs are observed at higher 8–10 CpG/100 bp density associated with CpG islands^50^.

A principal component analysis (PCA) of the DMRs for the control and case comparison for each group are presented in Supplemental Figure S1. Generally, the case and control DMR principal component 1 and 2 separated samples by treatment group, Supplemental Figure S1A–D. The African American samples circled generally clustered with the appropriate case or control groups, Supplemental Figure S1. Therefore, the racial background did not appear to have major impacts. As previously mentioned, the various clinical parameters in Supplemental Table S1b did not correlate with outliers in the PCA analysis, Supplemental Figure S1. Therefore, the DMRs identified appear to reflect PTB rather than specific pathology parameters or race.

A blinded test set of samples were collected to help validate the predictive ability of the PTB samples identified. Five triads for control and five triads for PTB case were collected for analysis. These samples were blinded to the WSU investigators during the analysis and prediction. This test set was analyzed and the data used in dendrogram, machine learning and PCA analysis, as previously described^44^. The accuracy for the test set mother was 50%, father was 40%, and female child 60%. However, after the analyses of the unblinded samples, a very heterogenous equal mixture of moderate, very, and extreme PTB were present. In addition, some batch effects within the assay were detected. Due to the low sample size (n = 5) of the test set and heterogeneity of the samples, this blinded test set analysis was potentially compromised and marginally successful, so not utilized for further analysis. As now discussed in the Discussion section, expanded clinical trials with larger sample size and larger test sample size are required to optimize and validate the epigenetic biomarkers (DMRs) identified.

**Figure 4:**
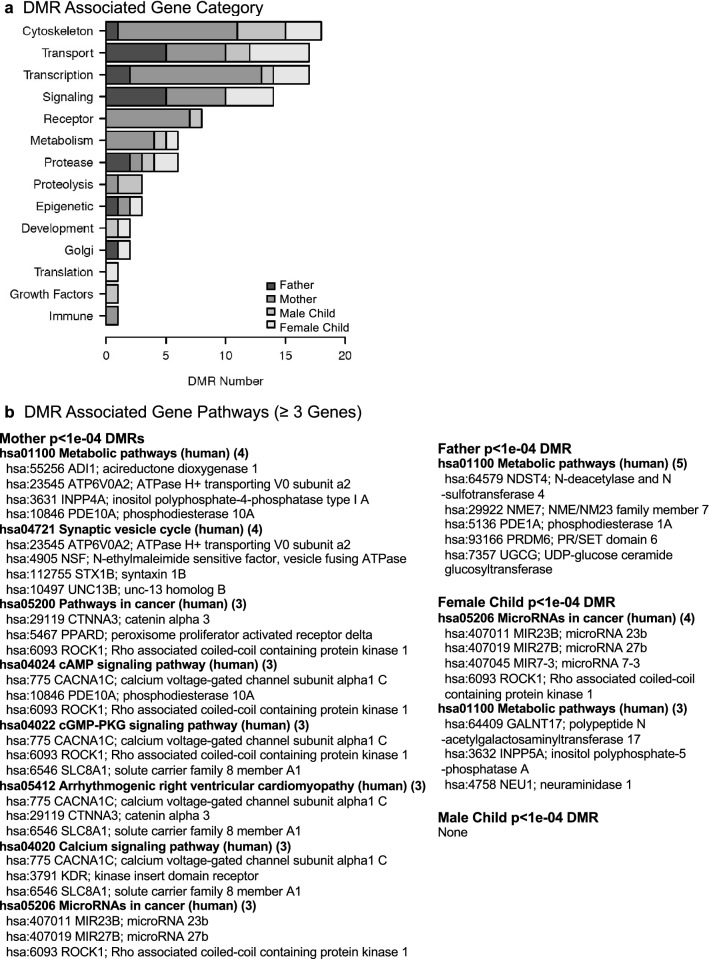
DMR gene associations (**a**) DMR (*p* < 1e−04) associated gene function category frequency. (**b**) DMR associated gene pathways for mother, father, and female child.

The final analysis investigated the DMR associated genes with each mother, father, and child DMR sets. The DMRs within 10 kb of a gene were considered to include proximal and distal promoter regions, as well as the gene. The DMR associated genes listed in Supplemental Tables S2–S5 were identified for gene functional category, Fig. 4a. The cytoskeleton, transport, transcription, and signaling categories were prominent in each group. The DMR associated gene groups were analyzed for KEGG pathways with ≥ 3 genes in the pathway, and the pathways and genes presented for each group, Fig. 4b. The mother DMR associated genes had the highest number of pathways with metabolism, synaptic vesicle cycle, and a number of signaling pathways prominent. The father had metabolism pathway, and male child no pathways. Interestingly, both the mother and female child had microRNA pathways represented (highlighted), Fig. 4b. This reflects DMRs shared between them that contain a cluster of genes and non-coding RNA, including Aopep (aminopeptidase O) and the micro-RNAs Mir 24-1, Mir 27b, Mir 23b, and Mir 3074. Therefore, an additional epigenetic mechanism altered in preterm birth appears to involve ncRNA that was common between the mother and daughter DMRs.

**Figure 5:**
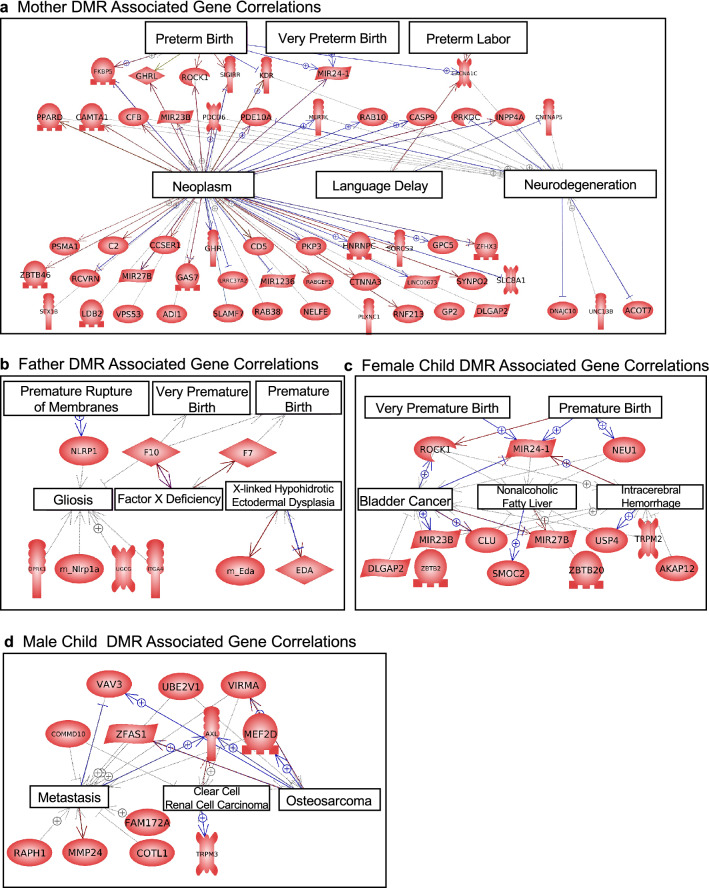
Associated gene networks and correlations. (**a**) Mother DMR associated gene correlations. (**b**) Father DMR associated gene correlations. The gene correlations and associated genes are presented for each disease group. (**c**) Female child DMR associated gene correlations. (**d**) Male child DMR associated gene correlations. The gene correlations and associated genes are presented for each disease pathology.

A presentation of the mother, father, and child DMR associated genes with network links, as determined by Pathway Studio (Elsevier, Inc.), are presented in Fig. 5. For each group the three disease states most over-represented in the list of DMR-associated genes are presented. Also included are any DMR associated genes with known associations with disease terms Premature Birth, Very Premature Birth, Preterm Labor, and Premature Rupture of Membranes. The mother, father, and female child groups all had DMR-associated genes previously shown to be linked to preterm birth. These known genes include Rock1, Ghrl1, Fkbp5, Sigirr, Kdr, Mir24-1, Cacna1c, Neu1, Nlrp1, F7 and F10, Fig. 5. This helps validate the potential PTB DMR biomarkers identified, as well as identify potential new DMRs and associated genes for PTB to consider.

## Discussion

Preterm birth is a major health concern worldwide, affecting more than one in 10 pregnancies^1^. Even when preterm children survive, they are at higher risk of developing chronic disease conditions^3–5^. These include hypertension, diabetes, metabolic and lipid disorders, heart disease, kidney disease, sleep apnea, and all cause mortality^5^. This is in part due to the stresses placed on the late-stage fetus, impacting their normal development. These impacts are studied in light of the Developmental Origins of Health and Disease (DOHAD) hypothesis. Previous studies have correlated many adult-onset diseases with fetal and early life developmental stresses^52–54^. The potential to predict preterm birth, and provide interventions to reduce its incidence, would have a significant impact on human health.

In this study buccal swab samples were collected from mothers, fathers and newborn infants approximately nine days following birth in cases where preterm birth occurred, and similarly in control full-term births. The buccal epithelial cells were analyzed for sites of DNA methylation in genomic regions when differences in methylation (DMRs) were detected. Mothers, fathers, and children all showed DMR signatures related to preterm birth (Figs. 1, 2). Male children had negligible DMR and a lower false discovery rate confidence than the other groups. The results of this study suggest that potential epigenetic tests of mothers, as well as fathers, could help predict the risk of preterm birth. However, extended prospective longitudinal pre-conception trials are required to optimize the potential biomarkers and assess the associations with different clinical parameters for preterm birth such as preeclampsia or obesity. Although the infant buccal analyses are not predictive of PTB, the epigenetic differences seen in children who have experienced preterm birth could potentially be used to assess later life disease (e.g., preterm birth) susceptibility and improve future preventative clinical management approaches. While it has been reported that paternal exposure to phenols is associated with increased incidence of preterm birth^55^, most previous studies have found that paternal lifestyle factors do not predict gestation length^56^. The current study identified epigenetic changes in both the mothers and fathers of children born preterm, suggesting potential maternal and paternal epigenetic components. Future expanded epigenetic analysis applied to both the mother and father may better assess risk of preterm birth, compared to assays of the mother alone.

The statistical confidence and accuracy of the prediction needs to be improved with expanded clinical trials with larger numbers of samples and trials monitoring individuals prior to conception of the child. Although, the current study demonstrates that epigenetic biomarkers in maternal and paternal buccal cells may be useful, larger studies are needed for predicting preterm birth. In the current study, buccal samples were collected from mothers and fathers immediately after the birth of their child. In the future, prospective studies with sample collection during pregnancy and prior to birth will be needed in order to develop a more clinically relevant predictive assay. Although a prospective study is anticipated to have similar DMR biomarkers, this remains to be confirmed.

In considering the accuracy of the epigenetic biomarkers observed, it is important to optimize with expanded clinical trials that include subpopulations of various sources of PTB such as obesity or preeclampsia. Interestingly some major disease biomarkers work approximately within a 50% accuracy range with either false positives or false negatives to consider. For example, for the major male prostate disease biomarker of Prostate Specific Antigen (PSA) for prostate cancer, the standard PSA cut-off of 4 ng/mL has low sensitivity. With this cut-off only 20.5% of the prostate cancer cases test positive and nearly 80% of prostate cancer cases are missed. The specificity at this cut-off is high (93.6%), meaning only 6.2% of men who do not have prostate cancer falsely test positive^57^. Another example is the ovarian cancer Ca125 biomarker which has a low accuracy for screening with both false positives and false negatives being problematic. However, for both PSA and Ca125, monitoring an individual over time does improve the accuracy of the assay to over 70% for monitoring, but not screening^58,59^. In addition, use of additional biomarkers in concert with the PSA and Ca125 has been found to improve the accuracy of screening to approximately 50%^60^. Due to the general low accuracy of such disease biomarkers, there have been a number of qualification and verification parameters put in place to improve and allow greater discovery efforts to be made for disease biomarkers^61,62^. Clearly disease biomarkers are essential for future medicine, but the current major protein-based biomarkers developed have limited use for general screening due to low accuracy. The current study provides large numbers of unique epigenetic-based DMR sites throughout the genome, which appear to relate to preterm birth. This is a unique molecular approach that may improve biomarker development. The study’s observations are encouraging and support the concept that epigenetic biomarkers derived from surrogate marker cells may be used as a biomarker for preterm birth. However, like PSA and Ca125, further clinical trials are needed to refine and validate the use of epigenetic biomarkers to predict preterm birth.

Previous studies have attempted to identify changes in DNA methylation in pregnant women that could be used as biomarkers of preterm birth. Parets et al.^63^ collected peripheral blood leukocyte samples from African American women at the start of labor that delivered either preterm (24–34 weeks; n = 16) or at term (39–41 weeks; n = 24). DNA methylation was assessed using the HumanMethylation450 BeadChip by Illumina. No DNA methylation biomarkers for preterm birth were identified, but these researchers did report that there were many DNA methylation changes that were shared between mothers that delivered preterm and their infants^63^. In a larger study of African American women, Hong et al.^64^ collected peripheral blood leukocyte samples at the time of labor from 150 women who delivered preterm, and 150 who delivered at term. DNA methylation was assessed using the Illumina HumanOmni2.5-4v1 array. Forty-five DMR were identified, of which two were found to be retained in a follow-up replication analysis^64^. Knijnenburg et al.^65^ performed a study that evaluated genomic variants, gene expression and DNA methylation simultaneously in whole blood samples taken in the day or two after birth. Two hundred seventy preterm and five hundred twenty-one full term maternal samples were evaluated. DNA methylation was assessed using the Illumina Methylation 450K array. No genomic variants were associated with preterm birth. However, 215 differentially expressed genes and two DMRs were found to be associated with preterm birth. There were greater numbers of molecular differences associated with very early preterm birth (< 28 weeks of gestation). Analysis of the 44 cases of these very early births showed that 217 genetic variants, 838 differentially expressed genes and 811 DMRs were associated^65^. A combined approach like this that uses multiple types of biomarkers shows promise for developing accurate clinical assays to predict preterm birth in the future. As previously mentioned, a limitation of all these studies is the use of mixed cell populations, which can suggest the presence of an epigenetic change, but which is in fact due to alterations in cell population numbers^32,33^. Purified individual cell types are more effective to both identify and assess epigenetic differences as disease biomarkers^41,42^.

A number of the previous studies have used the Illumina array platform to identify DMRs as biomarkers of preterm birth^63–65^. These array platforms are biased toward detection of DMR in high density CpG islands, which constitute less than 1% of the genome^50^. However, the majority of the genome has a low density (1–3%) 1–3 CpG/100 bp density ^50^. The MeDIP procedure used in the current study is biased toward detection of DNA methylation in regions of lower CpG density of < 5 CpG/100 bp, which corresponds to > 95% of the genome^50^. Using the genome-wide MeDIP procedure to identify DNA methylation alterations increases the feasibility of finding robust epigenetic biomarkers of preterm birth.

In the current study, only approximately half of the DMRs had nearby associated genes. Although the regulatory role of the DMRs to alter gene expression requires further investigation, the potential functional correlations of the DMR-associated genes for each group were evaluated. Genes involved in cytoskeleton, transcription and signaling were prominent in the gene sets (Figs. 4, 5). Among the disease states associated with these gene sets, the cancer pathways were frequently seen, possibly due to signaling abnormalities being prominent in cancer pathways. The mother, father and female child gene sets included DMR associated genes that have been previously associated with preterm birth (Fig. 5). This occurred even though cheek buccal cells are not directly involved in gestation, which suggests surrogate marker cell samples can be useful to detect epigenetic biomarkers of disease. This is supported by a recent study that used buccal cells to identify epigenetic biomarkers for female rheumatoid arthritis^46^.

## Conclusions

In conclusion, genome-wide differential DNA methylation regions for preterm birth were detected in buccal cells of mothers, fathers, and female children. This provides a “proof of concept” that DNA methylation analysis of buccal swabs of parents may be used to potentially predict preterm birth. However, the accuracy and predictive ability of the biomarker needs to be improved with future clinical trials, as discussed. Such a preterm birth risk or susceptibility biomarker would allow for better obstetrical management to prevent preterm birth, mitigate morbidity in unprevented preterm births (through timely administration of prenatal steroids, magnesium sulfate, tocolytics and optimal delivery procedures), and thus improve the health and long-term outcomes for many children. Unanticipated preterm births continue to catch providers by surprise, and often lead to major morbidities such as intraventricular hemorrhage, severe lung disease and other irreversible injuries. The presence of preterm birth associated DMRs in parental buccal cells suggests potential parental early life exposures and/or ancestral impacts are involved in the etiology of preterm birth. Rodent models have shown that environmental exposures in early pregnancy when epigenetic programming occurs in the fetus impact DMRs in every somatic cell type in the body across the life span of the exposed fetus and its descendants. Parents’ buccal cells, thus, may have the epigenetic changes resulting from ancestral exposure and can potentially be used as biomarkers for risk of preterm birth. This assay could also potentially be used in the future to identify environmental exposures and risk factors that promote preterm birth.

## Methods

### Clinical sample collection and analysis

St. Franciscan Hospital and Indiana University School of Medicine. IU Health Hospitals (Riley Hospital for Children, IUH Methodist, IUH North) and Franciscan Health, Indianapolis, Indiana, USA provided samples for the current study. Informed consent and HIPAA authorization was obtained from all participants prior to the clinical sample collection. The study protocol was approved by the Indiana University Institutional Review Board (IRB) #1901985132 and the Franciscan Institutional Review Board (IRB), #1489434-5. All research was performed in accordance with relevant guidelines/regulations. Informed consent and HIPAA authorization was obtained from all participants prior to sample collection. For sample collection involving human participants that are minors, informed consent from a parent and/or legal guardian for study participation was obtained prior to sample collection. Buccal samples were collected from the mother, father, and child in instances where pre-term birth occurred (case), or where term birth occurred (control), approximately nine days following birth. This period was used to allow the case PTB child to mature and allow and effective buccal cell collection. The demographic data for these subjects is presented in Supplemental Table S1. Buccal swabs were stored at -80 C until use.

### DNA preparation

Frozen human buccal samples were thawed for analysis. Genomic DNA from buccal samples was prepared as follows: The buccal brush was suspended in 750 μL of cell lysis solution and 3.5 µL of Proteinase K (20 mg/mL). This suspension was incubated at 55 ºC for 3 h, then vortexed and centrifuged briefly. The lysis solution was then transferred to a new 1.5 µL microcentrifuge tube. The microcentrifuge tube with the buccal brush was centrifuged again to retain any remaining solution which was combined with the transferred lysis solution. The buccal brush was discarded and 300 µL of protein precipitation solution (Promega, A795A, Madison, WI) was added to the lysis solution. The sample was incubated on ice for 15 min, then centrifuged at 4C for 30 min. The supernatant was transferred to a fresh 2 mL microcentrifuge tube and 1000 µL ice cold isopropanol was added along with 2 µL glycoblue. This suspension was mixed thoroughly and incubated at − 20 ºC overnight. The suspension was then centrifuged at 4ºC for 20 min, the supernatant was discarded, and the pellet was washed with 75% ethanol, then air-dried and resuspended in 100 μL H2O. DNA concentration was measured using the Nanodrop (Thermo Fisher, Waltham, MA).

### Methylated DNA immunoprecipitation (MeDIP)

Methylated DNA Immunoprecipitation (MeDIP) with genomic DNA was performed as follows: individual DNA samples (2–4 ug of total DNA) were diluted to 130 μL with 1 × Tris–EDTA (TE, 10 mM Tris, 1 mM EDTA) and sonicated with the Covaris M220 using the 300 bp setting. Fragment size was verified on a 2% E-gel agarose gel. The sonicated DNA was transferred from the Covaris tube to a 1.7 mL microfuge tube, and the volume was measured. The sonicated DNA was then diluted with TE buffer (10 mM Tris HCl, pH7.5; 1 mM EDTA) to 400 μL, heat-denatured for 10 min at 95 C, then immediately cooled on ice for 10 min. Then 100 μL of 5X IP buffer and 5 μg of antibody (monoclonal mouse anti 5-methyl cytidine; Diagenode #C15200006) were added to the denatured sonicated DNA. The DNA-antibody mixture was incubated overnight on a rotator at 4 C. The following day magnetic beads (Dynabeads M-280 Sheep anti-Mouse IgG; 11201D) were pre-washed as follows: The beads were resuspended in the vial, then the appropriate volume (50 μL per sample) was transferred to a microfuge tube. The same volume of Washing Buffer (at least 1 mL 1XPBS with 0.1% BSA and 2 mM EDTA) was added and the bead sample was resuspended. The tube was then placed into a magnetic rack for 1–2 min and the supernatant was discarded. The tube was removed from the magnetic rack and the beads were washed once. The washed beads were resuspended in the same volume of 1xIP buffer (50 mM sodium phosphate ph7.0, 700 mM NaCl, 0.25% TritonX-100) as the initial volume of beads. 50 μL of beads were added to the 500 μL of DNA-antibody mixture from the overnight incubation, then incubated for 2 h on a rotator at 4 C. After the incubation, the bead-antibody-DNA complex was washed three times with 1X IP buffer as follows: The tube was placed into a magnetic rack for 1–2 min and the supernatant was discarded, then the magnetic bead antibody pellet was washed with 1xIP buffer 3 times. The washed bead antibody DNA pellet was then resuspended in 250 μL digestion buffer with 3.5 μL Proteinase K (20 mg/mL). The sample was incubated for 2–3 h on a rotator at 55 C, then 250 μL of buffered Phenol–Chloroform- Isoamylalcohol solution was added to the sample, and the tube was vortexed for 30 s and then centrifuged at 14,000 rpm for 5 min at room temperature. The aqueous supernatant was carefully removed and transferred to a fresh microfuge tube. Then 250 μL chloroform were added to the supernatant from the previous step, vortexed for 30 s and centrifuged at 14,000 rpm for 5 min at room temperature. The aqueous supernatant was removed and transferred to a fresh microfuge tube. To the supernatant 2 μL of glycoblue (20 mg/mL), 20 μL of 5 M NaCl and 500 μL ethanol were added and mixed well, then precipitated in -20 C freezer for 1 h to overnight. The precipitate was centrifuged at 14,000 rpm for 20 min at 4 C and the supernatant was removed, while not disturbing the pellet. The pellet was washed with 500 μL cold 70% ethanol in − 20 C freezer for 15 min then centrifuged again at 14,000 rpm for 5 min at 4 C and the supernatant was discarded. The tube was spun again briefly to collect residual ethanol to the bottom of the tube and as much liquid as possible was removed with gel loading tip. The pellet was air-dried at RT until it looked dry (about 5 min) then resuspended in 20 μL H2O or TE. DNA concentration was measured in Qubit (Life Technologies) with ssDNA kit (Molecular Probes Q10212).

### MeDIP-Seq analysis

The MeDIP DNA samples (50 ng of each) were used to create libraries for next generation sequencing (NGS) using the NEBNext Ultra RNA Library Prep Kit for Illumina (San Diego, CA) starting at step 1.4 of the manufacturer’s protocol to generate double stranded DNA. After this step the manufacturer’s protocol was followed. Each sample received a separate index primer. NGS was performed at WSU Spokane Genomics Core using the Illumina HiSeq 2500 with a PE50 application, with a read size of approximately 50 bp and approximately 5–35 million reads per sample, and 6–7 sample libraries each were run in one lane.

### Molecular bioinformatics and statistics

Basic read quality was verified using information produced by the FastQC program^66^. Reads were filtered and trimmed to remove low quality base pairs using Trimmomatic^67^. The reads for each sample were mapped to the GRCh38 human genome using Bowtie2^68^ with default parameter options. The mapped read files were then converted to sorted BAM files using SAMtools^69^. To identify DMR, the reference genome was broken into 1000 bp windows. The MEDIPS R package^70^ was used to calculate differential coverage between control and exposure sample groups. The edgeR *p* value^71^ was used to determine the relative difference between the two groups for each genomic window. Windows with an edgeR p-value less than 10^–4^ were considered DMRs. The DMR edges were extended until no genomic window with an edgeR p-value less than 0.1 remained within 1000 bp of the DMR. CpG density and other information was then calculated for the DMR based on the reference genome. DMR were annotated using the NCBI provided annotations. The genes that overlapped with DMR were then input into the KEGG pathway search^72,73^ to identify associated pathways. The DMR associated genes were then sorted into functional groups by reducing Panther^74^ protein classifications into more general categories. All MeDIP-Seq genomic data obtained in the current study have been deposited in the NCBI public GEO database (GEO #: GSE194227).

Blinded test set analysis was performed to classify test samples into case or control groups. Samples from ten novel trios were collected to evaluate the efficacy of using the DMR sets identified as a biomarker for preterm birth. The test samples were processed identically to the samples used in the main analysis. PCA and cluster dendrogram analyses were used to search for test samples that clustered with the known samples when only DMR sites were considered. Additionally, linear discriminant analysis (LDA) and random forest (RF) classification was performed to identify which blinded samples were preterm birth, as previously described^44^.

### Ethics approval and consent to participate

Approvals to conduct the study were obtained from Indiana University Institutional Review Board (IRB) #1901985132 and the Franciscan Institutional Review Board (IRB), #1489434-5.

## Supplementary Information

Supplementary Legends.

Supplementary Figure 1.

Supplementary Figure 2.

Supplementary Figure 3.

Supplementary Table 1.

Supplementary Table 2.

Supplementary Table 3.

Supplementary Table 4.

Supplementary Table 5.

## Data Availability

All molecular data have been deposited into the public database at NCBI (GEO # GSE194227), and R code computational tools are available at GitHub (https://github.com/skinnerlab/MeDIP-seq) and www.skinner.wsu.edu.

## Abbreviations

EWAS: Epigenome-wide association study
DMRs: Differential DNA methylation regions
PTB: Preterm birth
ms-AFP: Maternal serum levels of alpha-fetoprotein
ms-hCG: Human chorionic gonadotropin
FSH: Follicle stimulating hormone
MeDIP: Methylated DNA immunoprecipitation
MeDIP-Seq: Methylated DNA immunoprecipitation followed by next generation sequencing
FT: Full term
FDR: False discovery rate
PCA: Principal component analysis
CTL: Control
Aopep: Aminopeptidase O
DOHAD: Developmental Origins of Health and Disease
PSA: Prostate Specific Antigen
