# Supplementary material for: Preterm birth buccal cell epigenetic biomarkers to facilitate preventative medicine
Source: Sci Rep. 2022 Mar 1;12:3361. doi: 10.1038/s41598-022-07262-9 (PMC8888575; doi:10.1038/s41598-022-07262-9)
Supplement: Supplementary file 1 — Supplementary Legends. [file 41598_2022_7262_MOESM1_ESM.pdf]

Supplemental Legends

Supplemental Figure S1. Principal component analysis (PCA). PCA with sample identification and the African American individuals circled in blue. **(A)** Mother PCA; **(B)** Father PCA; **(C)** Female child PCA; and **(D)** Male child PCA.

Supplemental Figure S2. DMR methylation log-fold change (X-axis) comparison with log 2p-value significance (Y-axis). Red indicates DMR individual's statistical significance ($p<1e-04$) and black not. **(A)** Mother; **(B)** Father; **(C)** Female child; and **(D)** Male child.

Supplemental Figure S3. DMR genomic features. **(A)** Father DMR CpG density; **(B)** Father DMR length; **(C)** Mother DMR CpG density; **(D)** Mother DMR length; **(E)** Male child DMR CpG density; **(F)** Male child DMR length; **(G)** Female child DMR CpG density; and **(H)** Female child DMR length.

Supplemental Table S1. Clinical sample information. **(A)** Sample information on mother, father and infant used in the analysis. **(B)** Clinical demographics for preterm birth and term birth samples with comparisons and statistical analysis presented.

Supplemental Table S2. Mother DMR list $p<1e-04$. DMR name, chromosome, start, stop, length, number significant windows, minimum p-value, max log-fold change, CpG number, CpG density, gene annotation, and gene category are presented.

Supplemental Table S3. Father DMR list p<1e-04. DMR name, chromosome, start, stop, length, number significant windows, minimum p-value, max log-fold change, CpG number, CpG density, gene annotation, and gene category are presented.

Supplemental Table S4. Female child DMR list p<1e-04. DMR name, chromosome, start, stop, length, number significant windows, minimum p-value, max log-fold change, CpG number, CpG density, gene annotation, and gene category are presented.

Supplemental Table S5. Male child DMR list p<1e-04. DMR name, chromosome, start, stop, length, number significant windows, minimum p-value, max log-fold change, CpG number, CpG density, gene annotation, and gene category are presented.
