# Supplementary material for: Preterm birth buccal cell epigenetic biomarkers to facilitate preventative medicine
Source: Sci Rep. 2022 Mar 1;12:3361. doi: 10.1038/s41598-022-07262-9 (PMC8888575; doi:10.1038/s41598-022-07262-9)
Supplement: Supplementary file 2 — Supplementary Figure 1. [file 41598_2022_7262_MOESM2_ESM.pdf]

Supplemental Figure S1

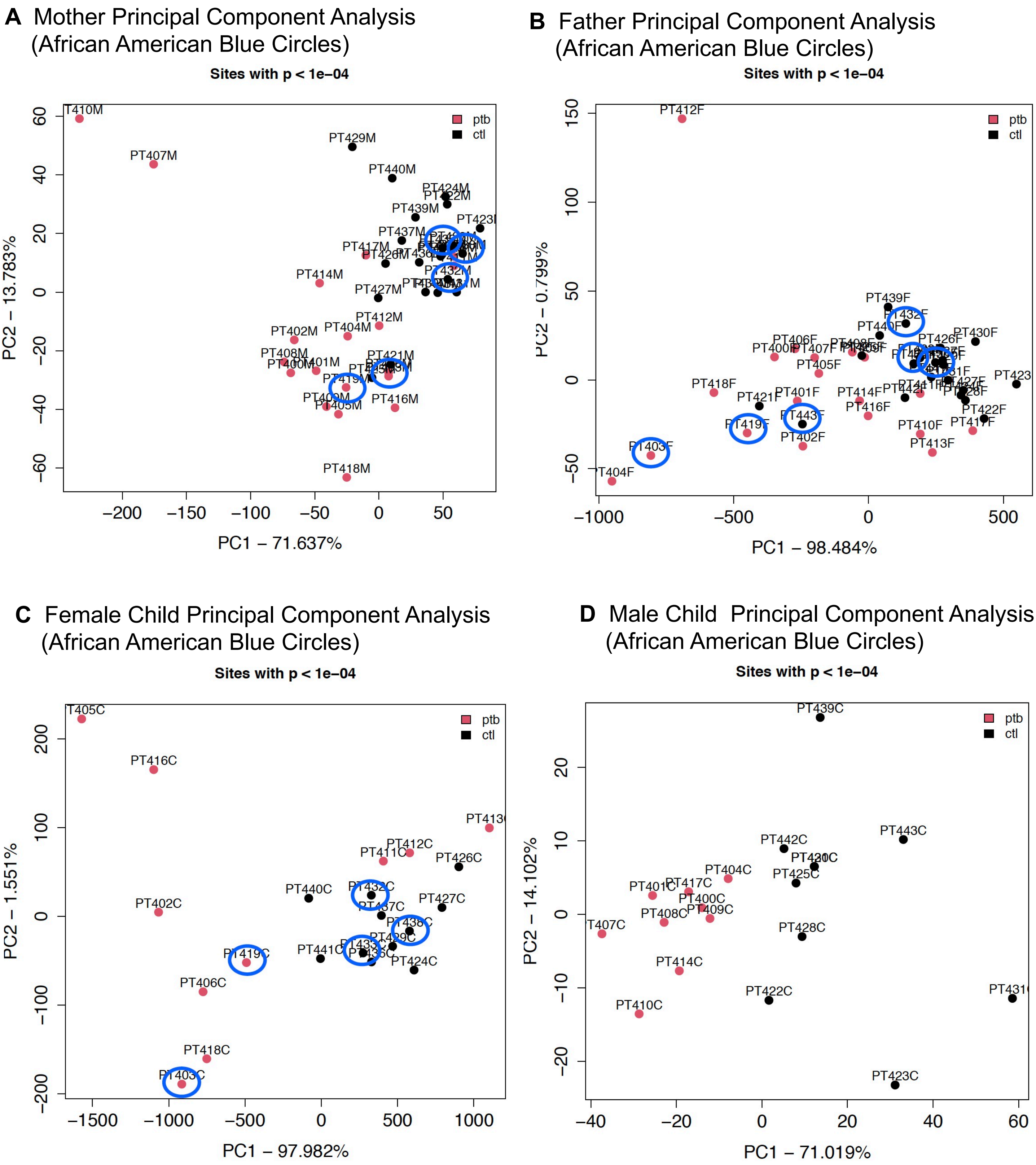

Supplemental Figure S1. Principal component analysis (PCA). PCA with sample identification and the African American individuals circled in blue. **(A)** Mother PCA; **(B)** Father PCA; **(C)** Female child PCA; and **(D)** Male child PCA.
