# Supplementary material for: Preterm birth buccal cell epigenetic biomarkers to facilitate preventative medicine
Source: Sci Rep. 2022 Mar 1;12:3361. doi: 10.1038/s41598-022-07262-9 (PMC8888575; doi:10.1038/s41598-022-07262-9)
Supplement: Supplementary file 3 — Supplementary Figure 2. [file 41598_2022_7262_MOESM3_ESM.pdf]

Supplemental Figure S2 DMR Log-Fold Change Correlations

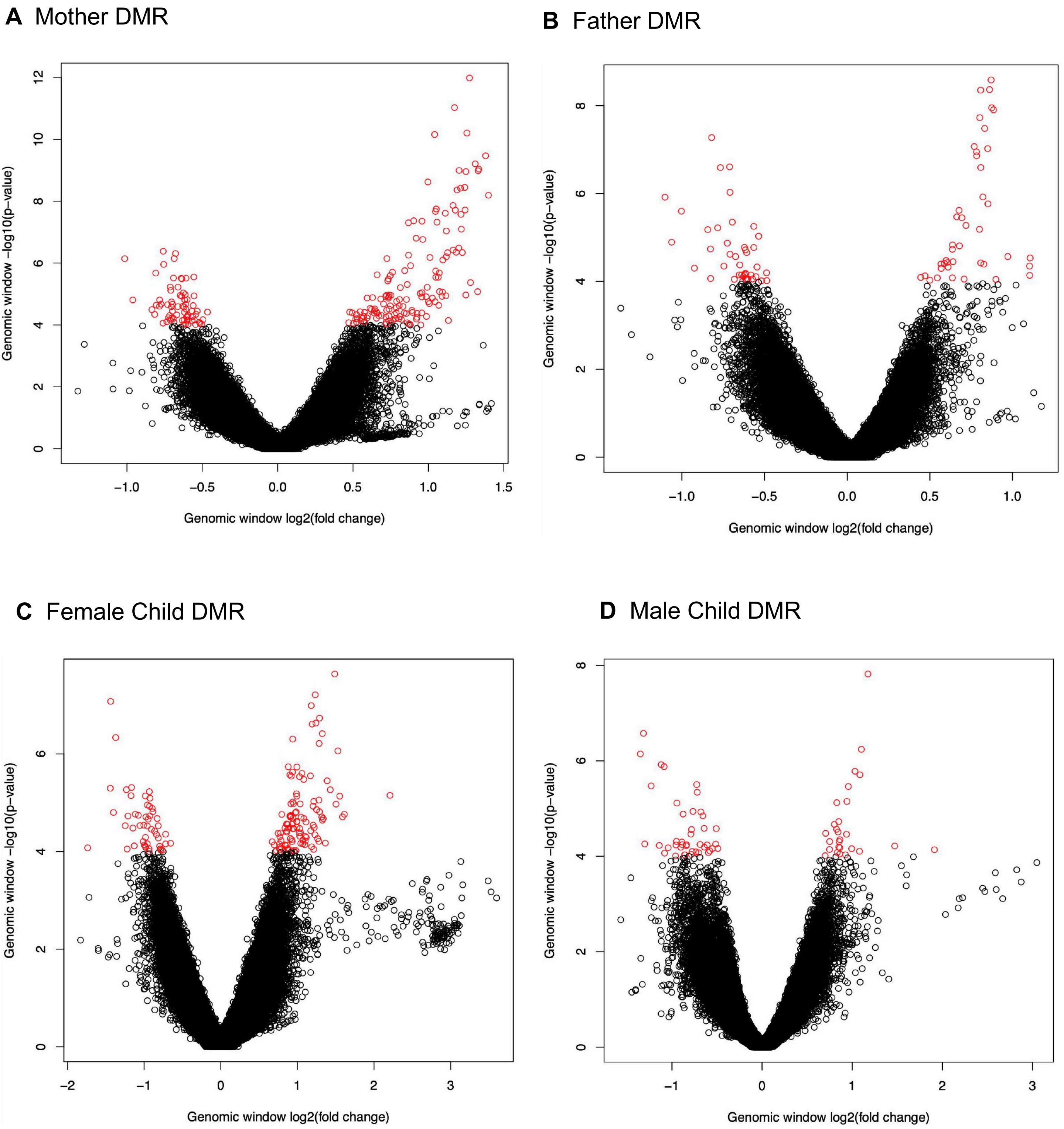

Supplemental Figure S2. DMR methylation log-fold change (X-axis) comparison with log 2p-value significance (Y-axis). Red indicates DMR individual's statistical significance (p<1e-04) and black not. **(A)** Mother; **(B)** Father; **(C)** Female child; and **(D)** Male child.
