# Supplementary material for: Preterm birth buccal cell epigenetic biomarkers to facilitate preventative medicine
Source: Sci Rep. 2022 Mar 1;12:3361. doi: 10.1038/s41598-022-07262-9 (PMC8888575; doi:10.1038/s41598-022-07262-9)
Supplement: Supplementary file 4 — Supplementary Figure 3. [file 41598_2022_7262_MOESM4_ESM.pdf]

Supplemental Figure S3 DMR Genomic Features

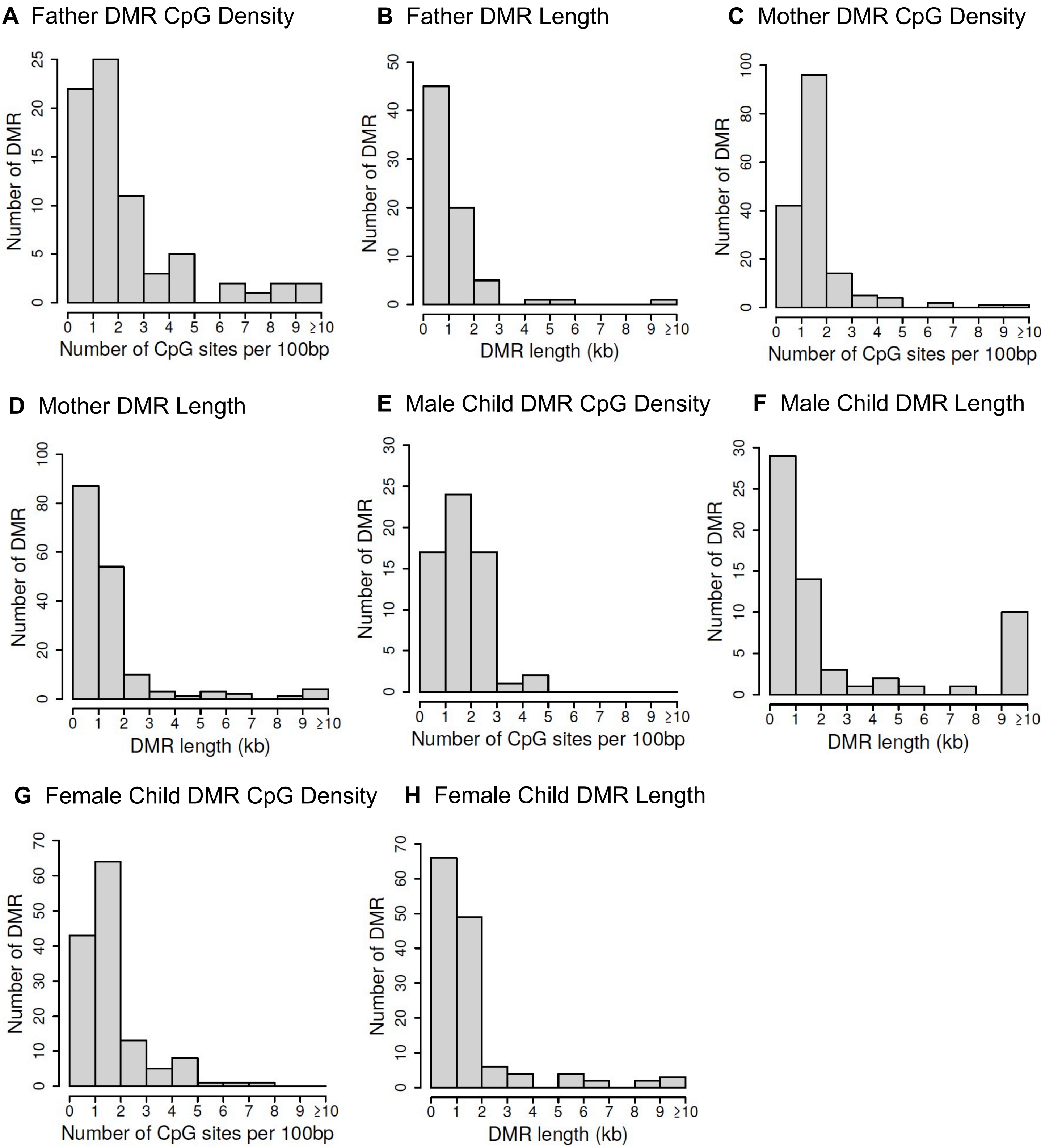

Supplemental Figure S3. DMR genomic features. **(A)** Father DMR CpG density; **(B)** Father DMR length; **(C)** Mother DMR CpG density; **(D)** Mother DMR length; **(E)** Male child DMR CpG density; **(F)** Male child DMR length; **(G)** Female child DMR CpG density; and **(H)** Female child DMR length.
