# Supplementary material for: Preterm birth buccal cell epigenetic biomarkers to facilitate preventative medicine
Source: Sci Rep. 2022 Mar 1;12:3361. doi: 10.1038/s41598-022-07262-9 (PMC8888575; doi:10.1038/s41598-022-07262-9)
Supplement: Supplementary file 5 — Supplementary Table 1. [file 41598_2022_7262_MOESM5_ESM.pdf]

Supplemental Table S1
(A) Clinical Sample Information

Kit #	Case or Control	Mother's Ethnicity & Race	Mother's Age at Enrollment	Father's Ethnicity & Race	Father's Age at Enrollment	Infant's Ethncity & Race	Infant Gender	Infant Category	Infant Age (weeks)	Infant Age	Year Enrolled
422	control	NH White	31	NH White	30	NH White	male	newborn	40	40 wks	2019
423	control	NH White	19	NH White	20	NH White	male	newborn	37	37 wks	2019
432	control	NH Black	20	NH Black	20	NH Black	female	newborn	38	38 wks	2020
436	control	NH White	28	NH White	28	NH White	female	newborn	39	39 wks	2019
421	control	NH White	38	NH White	39	NH White	male	newborn	38	38 wks	2019
437	control	NH White	26	NH White	27	NH White	female	newborn	40	40 wks	2019
440	control	NH White	33	NH White	37	NH White	female	newborn	38	38 wks	2019
442	control	NH White	22	NH White	24	NH White	male	newborn	38	38 wks	2019
425	control	NH White	27	NH White	29	NH White	male	newborn	41	41 wks	2019
424	control	NH White	31	NH White	33	NH White	female	newborn	38	38 wks	2019
428	control	NH White	23	NH White	41	NH White	male	newborn	39	39 wks	2020
439	control	NH White	34	NH White	38	NH White	male	newborn	39	39 wks	2019
426	control	NH White	37	NH White	38	NH White	female	newborn	39	39 wks	2020
430	control	NH White	32	NH White	31	NH White	male	newborn	39	39 wks	2020
427	control	NH White	27	NH White	29	NH White	female	newborn	37	37 wks	2020
441	control	NH White	31	NH White	33	NH White	female	newborn	39	39 wks	2019
429	control	NH White	30	NH white	31	NH White	female	newborn	39	39 wks	2020
438	control	NH Black & Nat	29	NH Black & Nat	32	NH Black & Nat	female	newborn	39	39 wks	2019
443	control	NH White	26	NH Black	25	NH White	male	newborn	39	39 wks	2020
431	control	NH White	29	NH White	34	NH White	male	newborn	39	39 wks	2020
433	control	NH Black	18	NH Black	19	NH Black	female	newborn	39	39 wks	2020
		mean ± SEM	28.1 ± 5.5		30.3 ± 6.3				38.7 ± 0.9		
400	PTB case	Hispanic White	31	NH White	41	NH White	male	newborn	32	32 wks	2019
401	PTB case	NH White	29	NH White	30	NH White	male	newborn	24	24 wks	2019
403	PTB case	NH Black	21	NH Black	24	NH Black	female	newborn	30	30 wks	2019
402	PTB case	NH White	34	NH White	29	NH White	female	newborn	31	31 wks	2019
404	PTB case	NH White	31	NH White	34	NH White	male	newborn	30	30 wks	2019
405	PTB case	NH White	34	NH White	37	NH White	female	newborn	30	30 wks	2019
406	PTB case	NH White	31	NH White	30	NH White	female	newborn	24	24 wks	2019
407	PTB case	NH White	25	NH White	29	NH White	male	newborn	32	32 wks	2019
408	PTB case	NH White & Nat	29	NH White & Nat	34	NH White & Nat	male	newborn	32	32 wks	2019
411	PTB case	NH White	34	NH White	38	NH White	female	newborn	31	31 wks	2019
409	PTB case	NH White	29	NH White	29	NH White	male	newborn	34	34 wks	2019
414	PTB case	NH White	28	NH White	31	NH White	Male	newborn	23	23 wks	2019
413	PTB case	NH White	27	NH White	25	NH White	female	newborn	30	30 wks	2019
410	PTB case	NH White	30	NH White	35	NH White	male	newborn	32	32 wks	2019
412	PTB case	NH White	25	NH White	28	NH White	female	newborn	33	33 wks	2019
416	PTB case	NH White	30	NH White	28	NH White	female	newborn	30	30 wks	2019
417	PTB case	NH White	26	Hispanic White	27	Hispanic White	male	newborn	34	34 wks	2019
418	PTB case	NH White	24	Unknown White	27	NH White	female	newborn	29	29 wks	2019
419	PTB case	NH Black	27	NH Black	29	NH Black	female	newborn	33	33 wks	2019
		mean ± SEM	28.7 ± 3.5		30.8 ± 4.5				30.2 ± 3.2		
Student T-Test - Case vs. Control			NS		NS				p<1e-03		

(B) Clinical Demographics

Variables	Preterm Birth Cases (n=19)	Term Birth Controls (n=21)	All Births (n=40)	p value
Parental Characteristics				
Maternal Age (years), mean (SD)	28.7 (3.54)	28.1 (5.51)	28.4 (4.63)	0.694
Paternal Age (years), mean (SD)	30.8 (4.54)	30.4 (6.38)	30.6 (5.51)	0.818
Maternal BMI at Delivery (kg/m^2), n (%)	33.0 (17.66)	32.9 (7.82)	33.0 (13.24)	0.976
Maternal BMI Categories, n (%)				
Normal weight (18.5-24.9)	6 (31.6%)	3 (14.3%)	9 (22.5%)	0.425
Overweight (25-29.9)	5 (26.3%)	7 (33.3%)	12 (30.0%)	
Obese (30 or greater)	7 (36.8%)	11 (52.4%)	18 (45.0%)	
Insurance, n (%)				
Medicaid/Self-Pay	10 (51.6%)	8 (38.1%)	18 (45.0%)	0.356
Private	9 (47.4%)	13 (61.9%)	22 (55.0%)	
Complications of Pregnancy				

Gravida, mean (min, max)	3.6 (1, 10)	1.7 (1, 5)	2.6 (1, 10)	0.014
Primiparous (first pregnancy), n (%)				
Yes	5 (26.3%	13 (61.9%)	18 (45.0%)	0.024
No	14 (73.7%)	8 (38.1%)	22 (55.0%)	
Multiparous with Prior Preterm Birth, n (%)				
Yes	6 (31.6%)	0 (0.0%)	6 (15.0%)	0.005
No	13 (68.4%)	21 (100.0%)	34 (85.0%)	
Multiparous with Prior Pregnancy Loss, n (%)				
Yes	8 (42.1%)	3 (14.3%	11 (27.5%)	0.049
No	11 (57.9%	18 (85.7%)	29 (72.5%)	
Substance Use (Tobacco, Alcohol, Drug Use), n (%)				
Current	11 (57.9%)	7 (33.3%)	18 (45.0%)	0.178
Former	2 (10.5%)	7 (33.3%)	9 (22.5%)	
None	5 (26.3%)	7 (33.3%)	12 (30.0%)	
Metabolic Disorder (Diabetes and/or Thyroid Disease), n (%)				
Yes	5 (26.5%)	4 (19.0%)	9 (22.5%)	0.455
No	12 (63.2%)	17 (81.0%)	29 (72.5 %)	
Pre-eclampsia, n (%)				
Yes	5 (26.3%)	1 (4.8%	6 (15.0%)	0.057
No	14 (73.7%)	20 (95.2%)	34 (85.0%)	
Obesity at End of Pregnancy, n (%)				
Yes	7 (36.8%)	11 (52.4%)	18 (45.0%)	0.399
No	11 (57.9%)	10 (47.6%)	21 (52.5%)	
Placenta Disorders (placenta previa/accreta/percreta, placenta abruption), n (%)				
Yes	4 (21.1%)	1 (4.8%)	5 (12.5%)	0.120
No	15 (78.9%)	20 (95.2%)	35 (87.5%)	
Cervical Insufficiency/Shortened Cervix, n (%)				
Yes	4 (21.1%)	0 (0.0%)	4 (10.0%)	0.190
No	13 (68.4%)	21 (100.0%)	34 (85.0%)	
Maternal Infection (bacterial or viral infection including UTI and STD), n (%)				
Yes	10 (52.6%)	9 (42.9%)	19 (47.5%)	0.194
No	7 (36.8%)	12 (57.1%)	19 (47.5%)	
Neuropsychological Disorders (anxiety, depression, ADHD, bipolar, schizophrenia, epilepsy, migraines), n (%)				
Yes	12 (63.2%)	9 (42.9%)	21 (52.5%)	0.240
No	5 (26.3%)	12 (57.1%)	17 (42.5%)	
Labor & Delivery Complications				
Medical Indicated Delivery, n (%)				
Yes	15 (78.9%)	8 (38.1%)	23 (57.5%)	0.009
No	4 (21.1%)	13 (61.9%)	17 (42.5%)	
Fetal Distress, n (%)				
Yes	5 (26.3%)	1 (4.8%)	6 (15.0%)	0.057
No	14 (73.7%)	20 (95.2%)	34 (85.0%)	
Meconium Stained Amniotic Fluid (MSAF), n (%)				
Yes	3 (15.8%)	8 (38.1%)	11 (27.5%)	0.190
No	15 (78.9%)	13 (61.9%)	28 (70.0%)	
Infant Characteristics				
Infant ethnicity/race, n (%)				
Hispanic	1 (5.3%)	0 (0.0%)	1 (2.5%)	0.746
Non-Hispanic White	15 (78.9%)	17 (81.0%)	32 (80.0%)	
Non-Hispanic Black	2 (10.5%)	3 (14.3%)	5 (12.5%)	
Non-Hispanic Other	1 (5.3%)	1 (4.8%)	2 (5.0%)	
Infant gender, n (%)				
Female	10 (47.6%)	11 (52.4%)	21 (52.5%)	0.987
Male	9 (52.4%)	10 (47.6%)	19 (47.5%)	
Birth Gestation (days), mean (SD)	214 (22.68)	274 (5.77)	245 (34.30)	<0.001
Birth Gestation (weeks), mean (min, max)	30.2 (23, 34)	38.8 (37, 41)	34.7 (23, 41)	<0.001
Birth Weight (grams), mean (SD)	1568 (577.07)	3333 (593.17)	[illegible]495 (1063.53)	<0.001
Birth Weight Percentile, mean (SD)	55.9 (24.74)	48.1 (31.57)	51.2 (28.45)	0.392
Fetal Growth, n (%)				
Small for Gestational Age (SGA)	2 (10.5%)	1 (4.8%)	3 (7.5%)	0.198
Appropriate for Gestational Age (AGA)	17 (89.5%)	17 (81.0)	34 (85.0%)	
Large for Gestational Age (LGA)	0 (0.0%)	3 (14.3)	3 (7.5%)	
5 Minute APGAR Scores, mean (min, max)	6 (2, 9)	9 (8, 9)	7 (2, 9)	<0.001
5 Minute APGAR Scores, n (%)				
Less than 5	8 (42.9%)	0 (0.0%)	8 (20%)	<0.001
5 or Greater	11 (57.9%)	21 (100.0%)	32 (80%)	
Hospital Length of Stay (days), mean (min, max)	52 (15, 124)	3 (2, 4)	27 (2, 124)	<0.001
