# Supplementary material for: Preterm birth buccal cell epigenetic biomarkers to facilitate preventative medicine
Source: Sci Rep. 2022 Mar 1;12:3361. doi: 10.1038/s41598-022-07262-9 (PMC8888575; doi:10.1038/s41598-022-07262-9)
Supplement: Supplementary file 6 — Supplementary Table 2. [file 41598_2022_7262_MOESM6_ESM.pdf]

Supplemetnal Table S2
DMR Table Mother 1e-04

DMR Name	Chr	Start	Length	# Sig Win	minP	minFDR	maxLFC	CpG #	CpG Density	Gene Annotation	Gene Category
1:4228001	1	4228001	1000	1	3.59E-05	5.28E-02	-0.569	32	3.2		
1:6308001	1	6308001	1000	1	5.30E-05	6.31E-02	-0.565	23	2.3	ACOT7	Metabolism
1:7006001	1	7006001	1000	1	3.16E-05	4.90E-02	0.623	5	0.5	CAMTA1	Transcription
1:34578001	1	34578001	1000	1	6.34E-05	6.99E-02	0.518	9	0.9	LOC105378641	
1:37091001	1	37091001	1000	1	5.01E-05	6.22E-02	0.598	11	1.1		
1:79942001	1	79942001	1000	1	5.82E-05	6.75E-02	-0.724	10	1		
1:90061001	1	90061001	1000	1	9.20E-06	2.43E-02	-0.626	15	1.5		
1:91213001	1	91213001	2000	1	2.59E-05	4.25E-02	-0.705	15	0.75		
1:122503001	1	122503001	1000	1	9.99E-05	9.18E-02	0.95	15	1.5		
1:124267001	1	124267001	1000	1	4.63E-07	2.48E-03	1.127	16	1.6		
1:143203001	1	143203001	3000	2	4.91E-06	1.50E-02	0.86	137	4.57		
1:143259001	1	143259001	2000	1	1.21E-06	5.12E-03	0.882	87	4.35		
1:143497001	1	143497001	2000	1	1.35E-05	2.89E-02	0.593	224	11.2	NKAIN1P1;LOC101927452;LINC02799	
1:160741001	1	160741001	1000	1	6.87E-05	7.33E-02	-0.553	9	0.9	SLAMF7	Immune
1:246575001	1	246575001	1000	1	7.49E-05	7.66E-02	0.552	17	1.7	TFB2M;CNST	Epigenetic
2:3503001	2	3503001	2000	1	6.82E-05	7.33E-02	-0.6	59	2.95	ADI1	Metabolism
2:26094001	2	26094001	1000	1	2.79E-05	4.50E-02	0.787	22	2.2	RAB10;LOC105374333;RPS2P15	
2:40496001	2	40496001	2000	2	1.93E-08	1.93E-04	1.243	9	0.45	SLC8A1	Transport
2:66702001	2	66702001	1000	1	1.08E-05	2.69E-02	0.74	10	1	LINC01798;LINC01797	
2:98567001	2	98567001	1000	1	6.18E-05	6.92E-02	0.528	13	1.3	INPP4A	
2:111970001	2	111970001	1000	1	4.93E-05	6.20E-02	-0.717	8	0.8	MERTK	Receptor
2:124257001	2	124257001	1000	1	5.30E-05	6.31E-02	0.571	8	0.8	CNTNAP5	
2:134653001	2	134653001	2000	1	2.13E-05	3.85E-02	-0.698	19	0.95	TMEM163	
2:179197001	2	179197001	3000	1	5.51E-05	6.51E-02	-0.605	23	0.77	SESTD1	
2:182748001	2	182748001	1000	1	7.10E-05	7.53E-02	-0.711	9	0.9	DNAJC10	Transcription
2:185552001	2	185552001	1000	1	4.17E-05	5.56E-02	0.925	6	0.6	ELF2P4	
3:27828001	3	27828001	1000	1	1.10E-05	2.69E-02	0.722	11	1.1	LINC01980;LINC01981	
3:75777001	3	75777001	3000	1	1.18E-05	2.74E-02	-0.638	67	2.23	ZNF717	Transcription
3:91542001	3	91542001	9000	3	1.81E-08	1.93E-04	1.052	137	1.52		
3:91552001	3	91552001	2000	2	6.94E-11	3.44E-06	1.041	32	1.6		
3:93322001	3	93322001	1000	1	1.23E-05	2.74E-02	0.985	17	1.7		
3:93630001	3	93630001	1000	1	1.75E-06	7.23E-03	0.843	19	1.9		
3:93706001	3	93706001	10000	7	4.40E-08	3.50E-04	0.958	170	1.7		
3:164339001	3	164339001	2000	1	1.45E-05	3.04E-02	0.684	36	1.8	LOC105374189	
3:195626001	3	195626001	2000	1	4.55E-05	5.91E-02	-0.816	58	2.9	MUC20P1;LOC107986029;LOC105374298	
4:16610001	4	16610001	1000	1	4.74E-05	6.03E-02	-0.596	13	1.3	LDB2	Transcription
4:34769001	4	34769001	1000	1	8.18E-05	8.12E-02	-0.688	26	2.6	LOC105378262	
4:40767001	4	40767001	1000	1	3.03E-06	1.02E-02	-0.691	7	0.7	NSUN7	
4:50475001	4	50475001	1000	1	1.03E-12	2.05E-07	1.273	15	1.5		
4:55077001	4	55077001	2000	1	6.15E-05	6.92E-02	0.56	7	0.35	KDR	Receptor
4:73538001	4	73538001	2000	1	1.97E-06	7.83E-03	0.762	18	0.9	LINC02499;LOC107986287	
4:90678001	4	90678001	2000	1	6.13E-05	6.92E-02	-0.692	19	0.95	CCSER1	
4:119064001	4	119064001	3000	1	8.15E-05	8.12E-02	-0.52	32	1.07	SYNPO2	Cytoskeleton
4:186128001	4	186128001	1000	1	9.68E-05	8.98E-02	-0.545	12	1.2	FAM149A	
5:290001	5	290001	1000	1	4.10E-05	5.54E-02	0.64	21	2.1	PDCD6-AHRR;PDCD6;LOC100421419	Cytoskeleton
5:42661001	5	42661001	2000	1	3.21E-05	4.94E-02	-0.835	13	0.65	GHR	Receptor
5:47308001	5	47308001	2000	1	4.09E-05	5.54E-02	0.956	35	1.75		
5:47691001	5	47691001	2000	1	2.04E-05	3.82E-02	1.006	40	2		
5:47753001	5	47753001	1000	1	5.62E-05	6.57E-02	0.924	16	1.6		
5:49072001	5	49072001	2000	1	1.76E-05	3.39E-02	1.001	36	1.8		
5:49609001	5	49609001	10000	3	4.29E-08	3.50E-04	0.902	156	1.56		

5:49621001	5	49621001	7000	3	2.14E-08	2.03E-04	1.047	117	1.67		
5:49630001	5	49630001	4000	2	3.33E-05	5.08E-02	0.775	48	1.2		
5:49642001	5	49642001	6000	3	1.77E-08	1.93E-04	1.179	96	1.6		
5:82206001	5	82206001	1000	1	9.47E-05	8.86E-02	-0.505	14	1.4	ATG10	Proteolysis
5:118384001	5	118384001	3000	1	3.05E-05	4.81E-02	-0.609	27	0.9	LINC02208	
5:120303001	5	120303001	1000	1	1.25E-05	2.75E-02	0.956	9	0.9		
6:25619001	6	25619001	1000	1	1.31E-05	2.85E-02	0.63	11	1.1	CARMIL1	
6:31946001	6	31946001	1000	1	7.37E-05	7.62E-02	-0.568	20	2	C2;C2-AS1;CFB;NELFE;MIR1236	
6:35335001	6	35335001	1000	1	1.52E-05	3.07E-02	-0.601	13	1.3	PPARD	Transcription
6:35625001	6	35625001	1000	1	1.22E-05	2.74E-02	0.737	33	3.3	FKBP5;LOC112267956	Transcription
6:44757001	6	44757001	1000	1	3.59E-05	5.28E-02	-0.698	5	0.5	LOC101929770	
6:46163001	6	46163001	1000	1	4.26E-05	5.64E-02	0.853	6	0.6	ENPP5	
6:59439001	6	59439001	1000	1	8.81E-05	8.53E-02	0.751	19	1.9		
6:59818001	6	59818001	2000	2	2.45E-08	2.21E-04	1.279	26	1.3		
6:120750001	6	120750001	1000	1	5.27E-05	6.31E-02	-0.564	8	0.8		
6:141567001	6	141567001	2000	1	8.90E-05	8.55E-02	-0.516	26	1.3		
6:160840001	6	160840001	19000	8	7.17E-07	3.26E-03	-0.756	305	1.61	LOC107986665	
6:165701001	6	165701001	2000	1	5.95E-06	1.77E-02	-0.713	29	1.45	PDE10A	Signaling
7:17809001	7	17809001	2000	1	4.16E-07	2.43E-03	-0.759	17	0.85	SNX13	Cytoskeleton
7:60991001	7	60991001	2000	1	3.95E-05	5.53E-02	0.576	133	6.65	LOC101060796	
7:90366001	7	90366001	1000	1	2.37E-05	4.12E-02	-0.561	7	0.7	GTPBP10	
7:117437001	7	117437001	1000	1	2.40E-05	4.12E-02	0.806	2	0.2	ASZ1;ANKRD49P4;LOC10537546 8	
7:153282001	7	153282001	1000	1	7.97E-08	5.65E-04	1.219	19	1.9	LOC102723686	
8:1255001	8	1255001	2000	1	4.91E-07	2.56E-03	-0.678	49	2.45	DLGAP2	Cytoskeleton
8:8567001	8	8567001	1000	1	3.84E-05	5.47E-02	-0.466	13	1.3	LOC105379224	
8:45500001	8	45500001	2000	2	1.04E-09	2.18E-05	1.399	29	1.45		
8:47791001	8	47791001	1000	1	7.88E-05	8.02E-02	-0.736	15	1.5	PRKDC	Signaling
8:139038001	8	139038001	1000	1	3.28E-06	1.05E-02	0.735	12	1.2		
9:24216001	9	24216001	2000	1	3.74E-05	5.46E-02	0.547	12	0.6		
9:35202001	9	35202001	1000	1	7.64E-06	2.17E-02	-0.709	11	1.1	UNC13B	
9:67907001	9	67907001	1000	1	3.95E-05	5.53E-02	0.894	4	0.4	ANKRD20A1;LOC644249	
9:95083001	9	95083001	1000	1	2.50E-05	4.21E-02	-0.784	42	4.2	AOPEP;MIR23B;MIR27B;MIR307 4;MIR24-1	Protease
10:41839001	10	41839001	3000	1	4.08E-05	5.54E-02	0.839	36	1.2		
10:66130001	10	66130001	1000	1	2.63E-05	4.28E-02	-0.621	15	1.5	CTNNA3;LOC105378340	Cytoskeleton
10:66638001	10	66638001	1000	1	6.04E-05	6.92E-02	0.655	12	1.2	CTNNA3	Cytoskeleton
10:104785001	10	104785001	1000	1	8.91E-05	8.55E-02	0.502	20	2	SORCS3	Transport
11:410001	11	410001	3000	1	7.14E-05	7.53E-02	1.133	86	2.87	PKP3;SIGIRR;ANO9	Cytoskeleton;Receptor
11:52128001	11	52128001	1000	1	2.33E-05	4.10E-02	1.068	11	1.1		
11:53986001	11	53986001	2000	2	8.40E-06	2.32E-02	1.327	22	1.1		
11:61099001	11	61099001	2000	1	2.10E-06	8.01E-03	-0.809	10	0.5	LOC105369325;CD5	
11:88030001	11	88030001	1000	1	1.56E-05	3.07E-02	-0.783	6	0.6	RAB38	
12:2177001	12	2177001	1000	1	2.22E-05	3.97E-02	-0.73	12	1.2	CACNA1C	Transport
12:3322001	12	3322001	2000	1	8.73E-05	8.50E-02	-0.761	26	1.3	LINC02827;LOC100128253;LOC1 00418939	
12:29017001	12	29017001	1000	1	6.20E-06	1.78E-02	0.814	7	0.7		
12:34829001	12	34829001	7000	2	1.01E-09	2.18E-05	1.249	110	1.57		
12:36739001	12	36739001	1000	1	2.88E-06	1.00E-02	1.049	14	1.4		
12:37007001	12	37007001	2000	1	6.61E-07	3.20E-03	1.01	26	1.3		
12:37245001	12	37245001	5000	1	3.77E-06	1.19E-02	0.73	76	1.52		
12:37259001	12	37259001	3000	1	1.37E-05	2.89E-02	0.735	34	1.13		
12:94221001	12	94221001	1000	1	2.91E-05	4.67E-02	0.613	9	0.9	PLXNC1	
12:107650001	12	107650001	2000	1	8.05E-05	8.07E-02	0.571	36	1.8	BTBD11	Cytoskeleton
12:123740001	12	123740001	2000	1	6.85E-05	7.33E-02	-0.621	30	1.5	ATP6V0A2	Metabolism
13:27422001	13	27422001	1000	1	1.05E-05	2.69E-02	-0.614	12	1.2	GTF3A	Transcription
13:37252001	13	37252001	2000	1	3.07E-06	1.02E-02	-0.609	16	0.8		
13:79890001	13	79890001	1000	1	5.31E-05	6.31E-02	-0.598	5	0.5	LINC00382	
13:84896001	13	84896001	1000	1	5.92E-05	6.83E-02	0.593	19	1.9	LOC105370290	
13:92819001	13	92819001	2000	1	7.17E-07	3.26E-03	0.725	14	0.7	GPC5	

13:97603001	13	97603001	1000	1	2.13E-05	3.85E-02	-0.774	7	0.7	LOC105370324	
14:30972001	14	30972001	1000	1	5.58E-05	6.56E-02	0.718	17	1.7	STRN3;HIGD1AP17	
15:19775001	15	19775001	6000	4	9.01E-06	2.42E-02	1.026	82	1.37		
15:75096001	15	75096001	1000	1	3.80E-05	5.47E-02	0.655	39	3.9		
16:20308001	16	20308001	1000	1	8.42E-05	8.27E-02	0.472	10	1	GP2	Receptor
16:22193001	16	22193001	2000	1	2.50E-05	4.21E-02	-0.631	29	1.45	SDR42E2;TRL-TAG3-1	Metabolism
16:31009001	16	31009001	1000	1	4.59E-05	5.91E-02	0.523	19	1.9	STX1B	Transcription
16:36668001	16	36668001	2000	2	4.31E-09	6.11E-05	1.191	43	2.15		
16:37462001	16	37462001	2000	2	3.38E-10	1.34E-05	1.38	35	1.75		
16:37955001	16	37955001	1000	1	3.39E-05	5.13E-02	0.891	19	1.9		
16:38168001	16	38168001	1000	1	6.30E-05	6.99E-02	0.804	16	1.6		
16:38265001	16	38265001	4000	4	2.38E-09	4.29E-05	1.119	68	1.7		
16:38275001	16	38275001	6000	3	1.11E-05	2.69E-02	0.751	85	1.42		
16:72850001	16	72850001	2000	1	4.06E-05	5.54E-02	0.495	32	1.6	ZFHX3	Transcription
16:88207001	16	88207001	1000	1	9.26E-05	8.80E-02	0.87	66	6.6		
17:642001	17	642001	2000	2	1.11E-05	2.69E-02	-0.647	46	2.3	VPS53	Transport
17:9897001	17	9897001	1000	1	3.05E-05	4.81E-02	0.645	21	2.1	GLP2R;RCVRN	Receptor
17:10004001	17	10004001	1000	1	3.85E-05	5.47E-02	0.477	22	2.2	GAS7	Cytoskeleton
17:46636001	17	46636001	2000	1	2.53E-05	4.22E-02	0.831	27	1.35	LRRC37A2;NSF	Receptor;Transport
17:72484001	17	72484001	1000	1	3.50E-05	5.26E-02	0.643	15	1.5	LINC00673	
17:80312001	17	80312001	1000	1	2.39E-06	8.96E-03	0.66	18	1.8	RNF213	
18:611001	18	611001	1000	1	8.04E-05	8.07E-02	0.523	7	0.7	CLUL1	
18:16382001	18	16382001	2000	2	2.87E-06	1.00E-02	1.065	27	1.35		
18:16431001	18	16431001	1000	1	3.77E-09	5.76E-05	1.214	12	1.2		
18:17126001	18	17126001	1000	1	1.80E-06	7.27E-03	1.048	14	1.4		
18:19182001	18	19182001	2000	2	3.81E-07	2.29E-03	1.184	32	1.6		
18:20561001	18	20561001	2000	1	9.45E-05	8.86E-02	0.877	29	1.45		
18:20937001	18	20937001	4000	2	2.67E-08	2.31E-04	1.216	54	1.35	ROCK1	Signaling
18:42878001	18	42878001	1000	1	1.21E-05	2.74E-02	0.716	6	0.6	RIT2	Signaling
18:78524001	18	78524001	1000	1	4.47E-05	5.88E-02	-0.543	15	1.5	LOC105372219	
19:7450001	19	7450001	2000	2	7.22E-07	3.26E-03	-1.015	74	3.7	ARHGEF18	
19:24963001	19	24963001	1000	1	1.19E-05	2.74E-02	1.091	16	1.6		
19:25291001	19	25291001	1000	1	7.93E-07	3.50E-03	1.096	19	1.9		
19:26672001	19	26672001	1000	1	8.70E-06	2.36E-02	1.094	16	1.6		
20:12359001	20	12359001	1000	1	3.25E-07	2.02E-03	1.202	7	0.7		
20:19932001	20	19932001	1000	1	4.16E-05	5.56E-02	0.661	13	1.3	RIN2	Transcription
20:26607001	20	26607001	2000	1	6.23E-11	3.44E-06	1.256	38	1.9		
20:27504001	20	27504001	2000	2	9.37E-12	9.30E-07	1.334	31	1.55		
20:27518001	20	27518001	2000	1	1.72E-07	1.10E-03	0.963	27	1.35		
20:63776001	20	63776001	2000	1	2.30E-05	4.08E-02	-0.806	72	3.6	ZBTB46	Cytoskeleton
21:8594001	21	8594001	2000	1	6.44E-05	7.06E-02	0.921	171	8.55		
21:9187001	21	9187001	1000	1	3.58E-05	5.28E-02	-0.534	12	1.2		
21:9330001	21	9330001	1000	1	6.73E-05	7.30E-02	-0.496	19	1.9	LOC101930100	
21:44523001	21	44523001	3000	1	2.81E-06	1.00E-02	-0.557	24	0.8	TSPEAR;TSPEAR-AS1;TSPEAR-AS2	Signaling
22:11968001	22	11968001	2000	1	3.24E-06	1.05E-02	-0.636	27	1.35		
22:12810001	22	12810001	1000	1	2.13E-05	3.85E-02	-0.61	15	1.5	LOC105379428	
22:50807001	22	50807001	2000	1	8.01E-05	8.07E-02	0.577	93	4.65	RPL23AP82	
X:1021001	X	1021001	1000	1	7.48E-05	7.66E-02	-0.601	11	1.1		
X:19143001	X	19143001	3000	1	1.15E-05	2.71E-02	-0.555	48	1.6		
X:30788001	X	30788001	19000	5	2.55E-05	4.23E-02	-0.706	131	0.69		
X:58735001	X	58735001	2000	2	4.57E-07	2.48E-03	1.225	31	1.55		
X:59064001	X	59064001	1000	1	8.37E-06	2.32E-02	1.074	18	1.8		
X:60549001	X	60549001	2000	2	3.57E-09	5.76E-05	1.241	28	1.4		
X:61211001	X	61211001	2000	1	1.77E-05	3.39E-02	1.044	37	1.85		
X:62067001	X	62067001	1000	1	1.14E-05	2.71E-02	1.116	17	1.7		
X:62486001	X	62486001	2000	1	6.09E-06	1.78E-02	0.992	42	2.1		
X:86590001	X	86590001	2000	1	4.01E-05	5.54E-02	0.786	23	1.15	DACH2	Transcription
