# Supplementary material for: Preterm birth buccal cell epigenetic biomarkers to facilitate preventative medicine
Source: Sci Rep. 2022 Mar 1;12:3361. doi: 10.1038/s41598-022-07262-9 (PMC8888575; doi:10.1038/s41598-022-07262-9)
Supplement: Supplementary file 7 — Supplementary Table 3. [file 41598_2022_7262_MOESM7_ESM.pdf]

Supplemental Table S3
DMR Table Father 1e-04

DMR Name	Chr	start	Length	# Sig Win	minP	minFDR	maxLFC	CpG #	CpG Density	Gene Annotation	Gene Category
1:10001	1	10001	1000	1	1.49E-05	4.63E-02	0.637	88	8.8	DDX11L1;WASH7P;MIR6859-1	
1:108206001	1	108206001	1000	1	1.69E-05	4.83E-02	-0.565	7	0.7	SLC25A24	Transport
1:169254001	1	169254001	1000	1	9.41E-07	6.04E-03	-0.709	10	1	NME7	Signaling
1:182237001	1	182237001	1000	1	9.99E-05	1.22E-01	-0.633	12	1.2	LINC01344	
2:87401001	2	87401001	5000	1	2.47E-07	1.76E-03	-0.711	119	2.38		
2:92270001	2	92270001	1000	1	4.48E-05	8.55E-02	1.103	14	1.4		
2:129414001	2	129414001	1000	1	8.76E-05	1.17E-01	-0.602	7	0.7		
2:181275001	2	181275001	1000	1	3.79E-05	8.11E-02	0.808	5	0.5	LINC01934	
2:182237001	2	182237001	1000	1	6.04E-06	2.30E-02	-0.784	18	1.8	PDE1A	Signaling
2:242183001	2	242183001	1000	1	2.41E-06	1.23E-02	0.677	21	2.1	RPL23AP88	
3:10001	3	10001	1000	1	5.05E-05	8.94E-02	0.569	15	1.5	LINC01986	
3:12080001	3	12080001	1000	1	8.65E-05	1.17E-01	-0.615	4	0.4	SYN2;ACTG1P12	Transport
3:91552001	3	91552001	2000	1	1.72E-06	9.30E-03	0.851	32	1.6		
3:161682001	3	161682001	1000	1	3.34E-05	7.63E-02	0.598	3	0.3		
3:174484001	3	174484001	1000	1	4.99E-05	8.94E-02	-0.925	28	2.8	NAALADL2	Protease
4:10001	4	10001	1000	1	6.53E-06	2.35E-02	0.8	120	12		
4:2826001	4	2826001	1000	1	6.50E-05	1.11E-01	-0.616	38	3.8	SH3BP2	
4:49166001	4	49166001	1000	1	9.59E-05	1.20E-01	-0.487	18	1.8	LOC101927209	
4:114937001	4	114937001	2000	1	3.56E-06	1.59E-02	0.694	13	0.65	NDST4	Transport
4:134832001	4	134832001	1000	1	1.35E-05	4.33E-02	-0.725	6	0.6		
4:188823001	4	188823001	2000	1	2.21E-05	5.67E-02	-0.609	47	2.35	LOC101930028	
4:190122001	4	190122001	2000	2	1.56E-05	4.70E-02	0.679	59	2.95	LOC107986338	
5:10001	5	10001	2000	2	1.12E-08	2.55E-04	0.885	6	0.3		
5:63075001	5	63075001	1000	1	3.88E-05	8.13E-02	0.588	6	0.6		
5:104719001	5	104719001	2000	1	7.10E-05	1.13E-01	-0.653	14	0.7	LOC105379109	
5:115622001	5	115622001	1000	1	3.40E-06	1.59E-02	0.663	10	1	TMED7-TICAM2;TICAM2-AS1;TMED7	Transport
5:123165001	5	123165001	1000	1	9.36E-05	1.19E-01	0.5	7	0.7	PRDM6	Transcription
5:124306001	5	124306001	1000	1	8.12E-05	1.17E-01	0.445	11	1.1	LINC01170;LOC107986448	
6:325001	6	325001	3000	1	1.83E-05	5.03E-02	-0.827	58	1.93	DUSP22	Signaling
6:160840001	6	160840001	6000	3	5.32E-08	6.83E-04	-0.82	99	1.65	LOC107986665	
6:160847001	6	160847001	11000	2	2.42E-05	6.06E-02	-0.709	187	1.7	LOC107986665	
7:10001	7	10001	1000	1	1.20E-06	6.93E-03	0.821	13	1.3	LOC102723872	
7:39010001	7	39010001	1000	1	9.23E-05	1.19E-01	-0.61	5	0.5	POU6F2;POU6F2-AS2	
7:50901001	7	50901001	1000	1	9.72E-05	1.20E-01	-0.519	6	0.6		
7:102564001	7	102564001	1000	1	7.16E-05	1.13E-01	-0.621	28	2.8	POLR2J3;SPDYE2	Transcription
8:53255001	8	53255001	1000	1	8.31E-05	1.17E-01	0.636	7	0.7	OPRK1	Signaling
8:85799001	8	85799001	3000	1	6.38E-05	1.11E-01	-0.488	40	1.33	REXO1L4P	
8:142563001	8	142563001	2000	1	1.92E-05	5.06E-02	-0.616	87	4.35	MROH4P	
9:25309001	9	25309001	1000	1	8.84E-05	1.17E-01	0.707	15	1.5		
9:111938001	9	111938001	1000	1	4.70E-05	8.66E-02	-0.546	14	1.4	UGCG;MIR4668	Golgi
10:10001	10	10001	1000	1	3.30E-08	4.84E-04	0.832	45	4.5	LOC102723376	
10:1932001	10	1932001	1000	1	4.22E-05	8.34E-02	-0.65	22	2.2		
10:97900001	10	97900001	2000	1	2.73E-05	6.52E-02	-0.675	40	2	CRTAC1	
11:53986001	11	53986001	2000	2	2.92E-05	6.83E-02	1.107	22	1.1		
11:117793001	11	117793001	2000	1	3.56E-05	7.95E-02	0.64	45	2.25	DSCAML1;LOC107984394	Cytoskeleton
12:10001	12	10001	1000	1	9.51E-08	9.78E-04	0.85	73	7.3	DDX11L8;WASH8P	Epigenetic
12:37263001	12	37263001	3000	1	4.05E-05	8.16E-02	0.827	47	1.57		
12:133264001	12	133264001	2000	2	2.60E-09	1.52E-04	0.871	49	2.45		
13:113111001	13	113111001	2000	1	7.79E-05	1.16E-01	-0.628	39	1.95	F7;F10	Protease
13:114354001	13	114354001	1000	1	4.43E-09	1.52E-04	0.807	2	0.2	LOC112268113	
16:846001	16	846001	2000	1	8.96E-05	1.17E-01	-0.683	88	4.4	LMF1	

16:15891001	16	15891001	1000	1	5.59E-06	2.21E-02	-0.565	18	1.8	CEP20	
17:642001	17	642001	2000	1	6.80E-05	1.13E-01	-0.615	46	2.3	VPS53	Transport
17:5511001	17	5511001	1000	1	1.68E-05	4.83E-02	-0.622	30	3	LOC728392;NLRP1;LOC101928044;RNU7-31P	
18:10001	18	10001	1000	1	3.72E-05	8.11E-02	0.607	38	3.8	LINC02564	
18:16431001	18	16431001	1000	1	9.01E-05	1.17E-01	0.899	12	1.2		
18:80262001	18	80262001	2000	2	1.88E-08	3.21E-04	0.802	92	4.6		
19:7450001	19	7450001	2000	2	2.52E-06	1.23E-02	-1.003	74	3.7	ARHGEF18	
19:33358001	19	33358001	1000	1	8.31E-05	1.17E-01	0.545	12	1.2	AKR1B1P7	
19:55850001	19	55850001	2000	2	1.21E-06	6.93E-03	-1.102	175	8.75	NLRP4	
19:58607001	19	58607001	1000	1	4.72E-05	8.66E-02	0.61	66	6.6	RPL23AP79	
20:12804001	20	12804001	1000	1	6.64E-06	2.35E-02	-0.844	7	0.7		
20:26607001	20	26607001	2000	1	2.72E-05	6.52E-02	0.971	38	1.9		
21:9329001	21	9329001	3000	1	7.82E-05	1.16E-01	-0.552	30	1	LOC101930100	
21:17751001	21	17751001	1000	1	7.15E-05	1.13E-01	-0.582	6	0.6	LOC105372741	
21:44523001	21	44523001	3000	1	9.37E-06	3.21E-02	-0.536	24	0.8	TSPEAR;TSPEAR-AS1;TSPEAR-AS2	Signaling
21:46699001	21	46699001	1000	1	4.02E-05	8.16E-02	0.568	109	10.9	RPL23AP4	
22:11968001	22	11968001	2000	1	7.11E-05	1.13E-01	-0.639	27	1.35		
22:50807001	22	50807001	2000	2	1.38E-07	1.18E-03	0.783	93	4.65	RPL23AP82	
X:222001	X	222001	1000	1	2.56E-07	1.76E-03	0.808	11	1.1		
X:427001	X	427001	1000	1	4.48E-06	1.92E-02	-0.695	67	6.7	LOC102724521	
X:69702001	X	69702001	1000	1	7.47E-05	1.15E-01	0.471	13	1.3	EDA	
X:156030001	X	156030001	1000	1	8.51E-08	9.71E-04	0.768	0	0	WASH6P;DDX11L16	
