# Supplementary material for: Preterm birth buccal cell epigenetic biomarkers to facilitate preventative medicine
Source: Sci Rep. 2022 Mar 1;12:3361. doi: 10.1038/s41598-022-07262-9 (PMC8888575; doi:10.1038/s41598-022-07262-9)
Supplement: Supplementary file 8 — Supplementary Table 4. [file 41598_2022_7262_MOESM8_ESM.pdf]

Supplemental Table S4
DMR Table Female Child 1e-04

DMR Name	Chr	Start	Length	# Sig Win	minP	minFDR	maxLFC	CpG #	CpG Density	Gene Annotation	Gene Category
6.292361111	1	9001	2000	1	4.17E-05	1.14E-01	0.781	88	4.4	DDX11L1;WASH7P;MIR6859-1	
1:77856001	1	77856001	2000	1	7.37E-05	1.52E-01	-0.997	21	1.05	MIGA1;NSRP1P1;HSPE1P25	
1:122462001	1	122462001	1000	1	9.41E-06	7.21E-02	1.214	20	2		
1:122885001	1	122885001	2000	1	9.03E-05	1.68E-01	1.212	41	2.05		
1:123674001	1	123674001	1000	1	9.60E-05	1.71E-01	0.981	17	1.7		
1:143259001	1	143259001	2000	1	2.48E-05	9.28E-02	0.992	87	4.35		
1:170269001	1	170269001	1000	1	5.00E-05	1.28E-01	0.771	6	0.6	LINC01142	
1:214113001	1	214113001	1000	1	6.31E-05	1.43E-01	0.671	10	1	LINC02775	
2:40496001	2	40496001	2000	1	2.29E-05	8.88E-02	1.326	9	0.45	SLC8A1	Transport
2:93921001	2	93921001	1000	1	8.68E-07	1.99E-02	1.528	13	1.3		
2:94809001	2	94809001	1000	1	1.55E-05	8.45E-02	0.982	9	0.9	ANKRD20A8P	
2:113624001	2	113624001	2000	1	2.00E-05	8.45E-02	0.912	20	1	RPL23AP7;RABL2A	
2:121547001	2	121547001	1000	1	2.95E-05	9.73E-02	-1.054	16	1.6	CLASP1	Cytoskeleton
2:185552001	2	185552001	1000	1	1.05E-05	7.22E-02	0.951	6	0.6	ELF2P4	
2:232742001	2	232742001	1000	1	4.24E-05	1.15E-01	0.749	7	0.7	GIGYF2	
2:241545001	2	241545001	2000	1	4.43E-05	1.18E-01	-0.72	40	2	BOK-AS1;LOC105373974	
3:49341001	3	49341001	1000	1	6.28E-05	1.43E-01	-1.012	40	4	USP4	Protease
3:91542001	3	91542001	9000	2	3.34E-06	4.38E-02	1.084	137	1.52		
3:91552001	3	91552001	2000	1	3.39E-05	1.01E-01	1.031	32	1.6		
3:93705001	3	93705001	11000	4	1.86E-06	3.66E-02	1.185	177	1.61		
3:114454001	3	114454001	1000	1	5.73E-05	1.37E-01	0.926	17	1.7	ZBTB20;ZBTB20-AS5	Transcription
3:135918001	3	135918001	2000	1	7.19E-06	6.16E-02	-1.171	14	0.7		
4:709001	4	709001	1000	1	1.54E-05	8.45E-02	-0.887	17	1.7	LOC107986245;PCGF3	Epigenetic
4:21928001	4	21928001	1000	1	2.74E-05	9.72E-02	0.858	4	0.4	KCNIP4	
4:49708001	4	49708001	1000	1	2.11E-05	8.45E-02	1.336	14	1.4		
4:50475001	4	50475001	1000	1	2.31E-08	6.36E-03	1.487	15	1.5		
4:104280001	4	104280001	1000	1	7.32E-05	1.52E-01	0.699	1	0.1	LOC105377350	
4:188823001	4	188823001	2000	1	6.95E-05	1.50E-01	-0.959	47	2.35	LOC101930028	
6.459027778	5	9001	3000	2	2.11E-05	8.45E-02	0.944	6	0.2		
5:15909001	5	15909001	2000	1	4.49E-05	1.19E-01	-0.839	19	0.95	FBXL7	
5:41287001	5	41287001	1000	1	1.84E-06	3.66E-02	0.881	11	1.1	LOC102723740	
5:47363001	5	47363001	1000	1	4.56E-05	1.20E-01	1.23	17	1.7		
5:47691001	5	47691001	2000	1	3.23E-05	1.01E-01	1.219	40	2		
5:49609001	5	49609001	10000	1	2.68E-06	4.04E-02	0.901	156	1.56		
5:49621001	5	49621001	7000	2	1.74E-05	8.45E-02	0.903	117	1.67		
5:49630001	5	49630001	4000	1	3.40E-05	1.01E-01	0.951	48	1.2		
5:49642001	5	49642001	6000	4	6.12E-07	1.53E-02	1.283	96	1.6		
5:117341001	5	117341001	1000	1	8.28E-05	1.62E-01	-0.98	6	0.6		
5:158184001	5	158184001	1000	1	1.31E-05	8.05E-02	-0.893	7	0.7	LOC100130177	
6:1046001	6	1046001	1000	1	5.43E-06	5.77E-02	-1.231	19	1.9	LINC01622	
6:11876001	6	11876001	1000	1	2.59E-05	9.50E-02	0.943	7	0.7	LOC102724379	
6:31850001	6	31850001	1000	1	8.86E-06	6.98E-02	1.281	18	1.8	NEU1	Metabolism
6:41613001	6	41613001	2000	1	4.90E-06	5.77E-02	-1.164	32	1.6		
6:59419001	6	59419001	1000	1	2.50E-06	4.04E-02	1.059	21	2.1		
6:96969001	6	96969001	1000	1	3.20E-05	1.01E-01	0.993	5	0.5	KLHL32	
6:151354001	6	151354001	1000	1	8.76E-05	1.67E-01	-0.936	27	2.7	AKAP12;ZBTB2	Cytoskeleton;Transcription
6:160847001	6	160847001	9000	1	3.84E-05	1.09E-01	-0.862	160	1.78	LOC107986665	
6:168613001	6	168613001	3000	2	8.41E-08	7.08E-03	-1.436	22	0.73	SMOC2	Signaling
7:10001	7	10001	1000	1	2.86E-06	4.04E-02	0.92	13	1.3	LOC102723872	
7:28233001	7	28233001	1000	1	1.61E-05	8.45E-02	0.973	6	0.6	JAZF1-AS1	
7:62509001	7	62509001	1000	1	9.72E-06	7.22E-02	0.939	17	1.7		
7:71679001	7	71679001	2000	2	7.36E-06	6.16E-02	1.552	20	1	GALNT17;RN7SKP75	Golgi
7:109796001	7	109796001	1000	1	7.84E-05	1.57E-01	-0.986	15	1.5		
8:1351001	8	1351001	2000	1	5.07E-06	5.77E-02	-1.441	104	5.2	DLGAP2	Cytoskeleton
8:45500001	8	45500001	2000	2	3.56E-06	4.46E-02	1.429	29	1.45		
9:18958001	9	18958001	2000	1	7.01E-05	1.50E-01	0.765	29	1.45	SAXO1	

9:42900001	9	42900001	1000	1	4.16E-05	1.14E-01	1.044	7	0.7	ANKRD20A7P;LOC112268044	
9:60601001	9	60601001	1000	1	9.73E-05	1.72E-01	0.926	11	1.1		
9:64123001	9	64123001	2000	2	2.49E-05	9.28E-02	1.114	10	0.5		
9:67404001	9	67404001	2000	1	2.93E-06	4.04E-02	1.028	21	1.05	LOC101927602	
9:95083001	9	95083001	1000	1	1.88E-05	8.45E-02	-1.21	42	4.2	AOPEP;MIR23B;MIR27B;MIR3074;MIR24-1	Protease
9:113088001	9	113088001	2000	1	8.43E-05	1.63E-01	-1.737	124	6.2		
9:132519001	9	132519001	2000	1	5.16E-05	1.30E-01	-0.982	17	0.85	CFAP77	Development
10:10001	10	10001	1000	1	5.47E-05	1.32E-01	0.906	45	4.5	LOC102723376	
10:49253001	10	49253001	2000	1	1.96E-05	8.45E-02	-0.976	11	0.55		
10:102726001	10	102726001	1000	1	9.82E-05	1.72E-01	0.823	13	1.3	SFXN2	Transport
10:132572001	10	132572001	3000	1	1.87E-05	8.45E-02	-0.925	83	2.77	INPP5A	Signaling
11:7759001	11	7759001	1000	1	9.11E-05	1.69E-01	0.769	4	0.4		
11:39308001	11	39308001	1000	1	6.23E-05	1.43E-01	0.932	6	0.6		
11:120219001	11	120219001	1000	1	1.72E-05	8.45E-02	-1.115	25	2.5	OAF	
11:135076001	11	135076001	1000	1	7.37E-05	1.52E-01	0.865	73	7.3	LINC02684	
12:29017001	12	29017001	1000	1	7.27E-05	1.52E-01	1.097	7	0.7		
12:34832001	12	34832001	4000	2	1.03E-07	7.08E-03	1.191	61	1.52		
12:37248001	12	37248001	2000	1	2.77E-05	9.72E-02	0.861	38	1.9		
12:37259001	12	37259001	3000	1	8.47E-05	1.63E-01	0.791	34	1.13		
12:45061001	12	45061001	1000	1	4.89E-05	1.26E-01	1.032	4	0.4	DBX2;RACGAP1P1;SSBL3P	
12:128665001	12	128665001	2000	1	2.98E-05	9.73E-02	-0.815	25	1.25	TMEM132C	
12:129867001	12	129867001	1000	1	7.67E-05	1.56E-01	-1.129	12	1.2	TMEM132D	
12:131580001	12	131580001	2000	1	2.96E-05	9.73E-02	-1.247	13	0.65	LOC105370084	
12:133264001	12	133264001	2000	2	2.76E-05	9.72E-02	0.931	49	2.45		
13:72001001	13	72001001	1000	1	9.53E-05	1.71E-01	0.724	8	0.8		
13:93878001	13	93878001	1000	1	1.98E-05	8.45E-02	1.048	6	0.6	GPC6	
13:114354001	13	114354001	1000	1	3.33E-05	1.01E-01	0.886	2	0.2	LOC112268113	
14:28184001	14	28184001	1000	1	6.11E-05	1.43E-01	1.102	7	0.7		
14:46509001	14	46509001	1000	1	4.97E-07	1.37E-02	0.939	18	1.8	LINC00871	
14:71081001	14	71081001	2000	1	7.28E-06	6.16E-02	-0.984	15	0.75	PCNX1;PTTG4P	
14:78984001	14	78984001	1000	1	6.59E-06	6.16E-02	0.988	6	0.6	NRXN3	
15:19775001	15	19775001	6000	1	1.06E-05	7.22E-02	1.036	82	1.37		
15:19782001	15	19782001	6000	1	9.63E-05	1.71E-01	0.957	88	1.47		
15:30739001	15	30739001	1000	1	9.93E-05	1.73E-01	-0.934	5	0.5	LOC100288637	
15:91263001	15	91263001	1000	1	5.29E-05	1.31E-01	-0.814	13	1.3	SV2B;LOC105370972	
15:98092001	15	98092001	1000	1	1.83E-05	8.45E-02	0.912	17	1.7	LINC01582	
16:36668001	16	36668001	2000	2	1.85E-07	9.71E-03	1.289	43	2.15		
16:37462001	16	37462001	2000	2	1.18E-05	7.43E-02	1.251	35	1.75		
16:37955001	16	37955001	1000	1	3.85E-07	1.33E-02	1.324	19	1.9		
16:38265001	16	38265001	4000	2	2.36E-06	4.04E-02	1.103	68	1.7		
16:38275001	16	38275001	6000	1	8.30E-05	1.62E-01	0.765	85	1.42		
16:55415001	16	55415001	1000	1	5.99E-05	1.41E-01	0.787	12	1.2		
16:88140001	16	88140001	1000	1	7.09E-06	6.16E-02	2.209	33	3.3		
17:642001	17	642001	2000	1	9.36E-05	1.71E-01	-0.783	46	2.3	VPS53	Transport
17:45576001	17	45576001	1000	1	1.07E-05	7.22E-02	1.504	7	0.7	DND1P1	
18:16430001	18	16430001	2000	1	1.53E-05	8.45E-02	1.289	20	1		
18:19182001	18	19182001	2000	2	1.73E-05	8.45E-02	1.612	32	1.6		
18:20937001	18	20937001	4000	1	6.19E-05	1.43E-01	1.28	54	1.35	ROCK1	Signaling
18:77407001	18	77407001	1000	1	5.94E-06	6.06E-02	-0.932	15	1.5	LOC107985171	
18:78126001	18	78126001	1000	1	1.19E-05	7.43E-02	-0.929	38	3.8		
18:79569001	18	79569001	1000	1	3.00E-05	9.73E-02	-0.958	28	2.8	LOC284240	
18:80262001	18	80262001	2000	2	2.79E-05	9.72E-02	0.874	92	4.6		
19:1170001	19	1170001	2000	1	4.64E-07	1.37E-02	-1.37	95	4.75	SBNO2;LOC102723798;LOC102725180	
19:4766001	19	4766001	1000	1	1.92E-05	8.45E-02	0.903	13	1.3	MIR7-3HG;MIR7-3	
19:7450001	19	7450001	2000	1	1.82E-05	8.45E-02	-1.075	74	3.7	ARHGEF18	
19:24908001	19	24908001	2000	1	5.45E-05	1.32E-01	1.264	35	1.75		
19:25098001	19	25098001	2000	1	6.82E-05	1.50E-01	1.043	37	1.85		
19:25291001	19	25291001	1000	1	2.89E-05	9.73E-02	1.206	19	1.9		
20:12359001	20	12359001	2000	1	6.17E-08	7.08E-03	1.232	13	0.65		

20:26599001	20	26599001	2000	1	6.74E-05	1.50E-01	1.364	38	1.9		
20:26607001	20	26607001	2000	1	3.75E-05	1.09E-01	1.174	38	1.9		
20:27504001	20	27504001	2000	2	1.96E-05	8.45E-02	1.318	31	1.55		
20:27518001	20	27518001	2000	1	6.96E-05	1.50E-01	1.07	27	1.35		
20:63061001	20	63061001	1000	1	1.12E-05	7.33E-02	-0.958	41	4.1	LINC01749;LINC01056	
21:9329001	21	9329001	3000	1	6.78E-05	1.50E-01	-0.654	30	1	LOC101930100	
21:17164001	21	17164001	1000	1	2.17E-05	8.53E-02	-0.946	16	1.6		
21:44404001	21	44404001	2000	1	8.90E-05	1.67E-01	-0.79	43	2.15	TRPM2;TRPM2-AS	Transport
21:45324001	21	45324001	1000	1	8.91E-05	1.67E-01	-1.222	22	2.2	LOC105372838	
22:11968001	22	11968001	2000	1	8.08E-06	6.55E-02	-0.924	27	1.35		
22:11974001	22	11974001	2000	1	2.89E-05	9.73E-02	-1.004	64	3.2		
22:50807001	22	50807001	2000	2	1.06E-05	7.22E-02	0.915	93	4.65	RPL23AP82	
X:9568001	X	9568001	2000	1	2.11E-05	8.45E-02	-0.83	34	1.7	TBL1X	
X:30788001	X	30788001	11000	1	3.50E-05	1.03E-01	-0.905	68	0.62		
X:30800001	X	30800001	7000	1	6.98E-05	1.50E-01	-0.719	57	0.81		
X:65379001	X	65379001	3000	1	7.80E-05	1.57E-01	-0.797	38	1.27	ZC3H12B	Translation
X:68213001	X	68213001	1000	1	3.30E-05	1.01E-01	0.838	11	1.1	OPHN1	Signaling
X:86590001	X	86590001	2000	1	5.80E-05	1.38E-01	1.072	23	1.15	DACH2	Transcription
X:119438001	X	119438001	2000	1	9.44E-05	1.71E-01	-0.764	19	0.95	SLC25A43;RN7SL118P	Transport
X:120874001	X	120874001	1000	1	3.31E-05	1.01E-01	-1.146	25	2.5	PA2G4P1;CT47B1;CT47A12	
X:156030001	X	156030001	1000	1	7.54E-05	1.54E-01	0.846	0	0	WASH6P;DDX11L16	
