# Supplementary material for: Preterm birth buccal cell epigenetic biomarkers to facilitate preventative medicine
Source: Sci Rep. 2022 Mar 1;12:3361. doi: 10.1038/s41598-022-07262-9 (PMC8888575; doi:10.1038/s41598-022-07262-9)
Supplement: Supplementary file 9 — Supplementary Table 5. [file 41598_2022_7262_MOESM9_ESM.pdf]

Supplemental Table S5
DMR Table Male Child 1e-04

DMR Name	Chr	Start	Length	# Sig Win	minP	minFDR	maxLFC	CpG #	CpG Density	Gene Annotation	Gene Category
1:107960001	1	107960001	1000	1	6.51E-05	1.99E-01	-0.902	12	1.2	VAV3;TRUND-NNN8-1;VAV3-AS1	
1:201075001	1	201075001	1000	1	2.73E-05	1.65E-01	0.826	18	1.8	CACNA1S	Transport
2:361001	2	361001	2000	1	1.33E-06	3.29E-02	-1.086	44	2.2	LOC105373351	
2:860001	2	860001	2000	2	7.31E-05	2.02E-01	1.912	36	1.8	LINC01115	
2:47667001	2	47667001	1000	1	4.88E-05	1.99E-01	-0.95	12	1.2		
2:203444001	2	203444001	1000	1	5.83E-05	1.99E-01	-0.955	17	1.7	RAPH1	Cytoskeleton
3:28671001	3	28671001	2000	1	1.88E-05	1.40E-01	0.85	14	0.7	LINC00693	
3:44344001	3	44344001	2000	1	6.43E-05	1.99E-01	0.851	26	1.3	TOPAZ1;LOC105377055;TCAIM	
3:129500001	3	129500001	1000	1	1.66E-06	3.53E-02	1.03	15	1.5	IFT122	
3:130629001	3	130629001	1000	1	7.97E-05	2.07E-01	-0.775	20	2	COL6A6	
4:131421001	4	131421001	1000	1	9.35E-05	2.08E-01	-0.859	10	1		
4:173099001	4	173099001	2000	1	8.61E-05	2.07E-01	-1.081	29	1.45		
5:94011001	5	94011001	2000	1	1.20E-06	3.29E-02	-1.119	30	1.5	FAM172A	
5:116157001	5	116157001	1000	1	5.94E-05	1.99E-01	-1.14	4	0.4	COMMD10	
5:139743001	5	139743001	1000	1	2.15E-05	1.53E-01	0.801	9	0.9	PSD2;PSD2-AS1	Transcription
5:157464001	5	157464001	1000	1	9.74E-05	2.13E-01	0.699	9	0.9	NIPAL4-DT;NIPAL4	
6:11964001	6	11964001	1000	1	3.46E-05	1.78E-01	0.936	7	0.7	LOC107986570	
6:21559001	6	21559001	2000	1	3.31E-05	1.78E-01	0.706	17	0.85		
6:50227001	6	50227001	1000	1	9.35E-05	2.08E-01	-0.856	5	0.5		
6:81469001	6	81469001	1000	1	6.05E-05	1.99E-01	-0.841	14	1.4	LOC105377871	
6:160853001	6	160853001	5000	1	8.13E-05	2.07E-01	-0.734	89	1.78	LOC107986665	
7:22571001	7	22571001	1000	1	7.21E-05	2.02E-01	0.748	10	1	LOC100506178	
9:71282001	9	71282001	1000	1	6.10E-05	1.99E-01	1.471	6	0.6	TRPM3;LOC107987079	Transport
10:5561001	10	5561001	2000	1	4.92E-05	1.99E-01	0.75	39	1.95	LOC105376380;LOC105376381	
10:38513001	10	38513001	17000	1	5.80E-05	1.99E-01	-0.609	419	2.46		
10:38808001	10	38808001	14000	1	9.21E-05	2.08E-01	-0.785	366	2.61		
10:38869001	10	38869001	37000	2	3.16E-06	4.71E-02	-0.725	941	2.54		
10:66130001	10	66130001	1000	1	9.88E-05	2.13E-01	-0.96	15	1.5	CTNNA3;LOC105378340	Cytoskeleton
10:99323001	10	99323001	1000	1	3.47E-06	4.71E-02	0.957	9	0.9	CNNM1	
10:105370001	10	105370001	1000	1	3.11E-05	1.78E-01	0.843	10	1		
12:52611001	12	52611001	1000	1	1.52E-08	2.27E-03	1.174	14	1.4	KRT72;KRT73;KRT73-AS1	
12:61942001	12	61942001	1000	1	5.74E-05	1.99E-01	-0.884	16	1.6	TAFA2	Growth Factors
12:111739001	12	111739001	1000	1	4.92E-05	1.99E-01	0.855	13	1.3	ACAD10	Metabolism
15:30844001	15	30844001	4000	1	7.19E-07	2.68E-02	-1.35	20	0.5		
15:84075001	15	84075001	1000	1	6.64E-05	1.99E-01	-1.041	6	0.6	EFL1P1	
16:84571001	16	84571001	1000	1	4.48E-05	1.99E-01	0.863	30	3	COTL1	Cytoskeleton
17:21851001	17	21851001	12000	1	2.76E-05	1.65E-01	-0.782	359	2.99		
17:21865001	17	21865001	74000	3	1.15E-05	1.04E-01	-0.79	2000	2.7		
17:26783001	17	26783001	44000	1	7.78E-05	2.07E-01	-0.579	1206	2.74		
17:26828001	17	26828001	17000	2	1.43E-05	1.12E-01	-0.687	472	2.78		
17:26846001	17	26846001	12000	1	8.46E-05	2.07E-01	-0.702	325	2.71		
17:31725001	17	31725001	1000	1	9.35E-05	2.08E-01	0.925	22	2.2		
19:309001	19	309001	2000	1	5.04E-05	1.99E-01	-0.89	86	4.3	MIER2	Development
19:2013001	19	2013001	2000	1	6.65E-05	1.99E-01	0.861	44	2.2	BTBD2;LOC107985278	Proteolysis
19:7450001	19	7450001	2000	2	2.66E-07	1.99E-02	-1.315	74	3.7	ARHGEF18	
19:35977001	19	35977001	1000	1	7.55E-06	7.65E-02	0.828	17	1.7		
19:41238001	19	41238001	2000	1	6.96E-05	1.99E-01	0.994	34	1.7	AXL;TRK-TTT10-1	Receptor
19:54472001	19	54472001	3000	1	1.96E-06	3.65E-02	1.085	38	1.27	LENG8;LENG9;CDC42EP5	Cytoskeleton
20:2443001	20	2443001	1000	1	7.70E-06	7.65E-02	-0.944	15	1.5		
20:31231001	20	31231001	6000	1	1.18E-05	1.04E-01	-0.678	144	2.4		
20:31238001	20	31238001	8000	1	8.40E-05	2.07E-01	-0.595	217	2.71		
20:35263001	20	35263001	1000	1	7.11E-06	7.65E-02	0.941	19	1.9	MMP24-AS1-EDEM2;MMP24	Protease
20:49291001	20	49291001	1000	1	8.97E-05	2.08E-01	0.862	8	0.8	ZFAS1	
20:50125001	20	50125001	2000	1	8.36E-05	2.07E-01	0.833	29	1.45	PEDS1-UBE2V1;UBE2V1;PEDS1	Proteolysis
21:9064001	21	9064001	2000	1	5.75E-07	2.68E-02	1.101	18	0.9	LOC101927615;TEKT4P2	

21:10665001	21	10665001	42000	2	2.64E-05	1.65E-01	-0.551	871	2.07		
22:10716001	22	10716001	15000	1	5.58E-05	1.99E-01	-0.515	336	2.24		
22:11968001	22	11968001	3000	1	1.31E-05	1.09E-01	-0.876	37	1.23		
22:15879001	22	15879001	3000	1	5.53E-05	1.99E-01	-1.301	122	4.07	NBEAP3	
22:47712001	22	47712001	1000	1	5.73E-05	1.99E-01	-0.839	8	0.8	LOC284930	
Y:56825001	Y	56825001	5000	2	4.57E-06	5.67E-02	-0.72	118	2.36		
